# Engineered cell culture microenvironments for mechanobiology studies of brain neural cells

**DOI:** 10.3389/fbioe.2022.1096054

**Published:** 2022-12-14

**Authors:** Lucía Castillo Ransanz, Pieter F. J. Van Altena, Vivi M. Heine, Angelo Accardo

**Affiliations:** ^1^ Department of Child and Adolescence Psychiatry, Amsterdam Neuroscience, Emma Children’s Hospital, Amsterdam UMC Location Vrije Universiteit Amsterdam, Amsterdam, Netherlands; ^2^ Department of Precision and Microsystems Engineering, Delft University of Technology, Delft, Netherlands; ^3^ Center for Neurogenomics and Cognitive Research, Vrije Universiteit Amsterdam, Amsterdam Neuroscience, Department of Complex Trait Genetics, Amsterdam UMC Location Vrije Universiteit Amsterdam, Amsterdam, Netherlands

**Keywords:** 3D scaffold, *in vitro* models, neurons, iPSC, organoids, mechanobiology, microfabrication, stem cells

## Abstract

The biomechanical properties of the brain microenvironment, which is composed of different neural cell types, the extracellular matrix, and blood vessels, are critical for normal brain development and neural functioning. Stiffness, viscoelasticity and spatial organization of brain tissue modulate proliferation, migration, differentiation, and cell function. However, the mechanical aspects of the neural microenvironment are largely ignored in current cell culture systems. Considering the high promises of human induced pluripotent stem cell- (iPSC-) based models for disease modelling and new treatment development, and in light of the physiological relevance of neuromechanobiological features, applications of *in vitro* engineered neuronal microenvironments should be explored thoroughly to develop more representative *in vitro* brain models. In this context, recently developed biomaterials in combination with micro- and nanofabrication techniques 1) allow investigating how mechanical properties affect neural cell development and functioning; 2) enable optimal cell microenvironment engineering strategies to advance neural cell models; and 3) provide a quantitative tool to assess changes in the neuromechanobiological properties of the brain microenvironment induced by pathology. In this review, we discuss the biological and engineering aspects involved in studying neuromechanobiology within scaffold-free and scaffold-based 2D and 3D iPSC-based brain models and approaches employing primary lineages (neural/glial), cell lines and other stem cells. Finally, we discuss future experimental directions of engineered microenvironments in neuroscience.

## Introduction

The brain is comprised of highly organized architectures (Sapir et al, 2022) that are subjected to and exercise various physical cues at different scales. At the nanoscale, for example, nanometric properties such as the composition and organization of the extracellular matrix provide cues to brain cells. These cues include mechanical stimuli, such as the forces exerted by different neural cell types, and topographical cues such as the biomechanical properties of the neural microenvironment (e.g., porosity, roughness, rigidity, viscoelasticity, etc.) (Yu et al, 2022). The resulting bidirectional physical interactions and the mechanobiology features in the brain microenvironment are translated into biochemical signals through mechanotransduction, subsequently eliciting various cellular responses to guide brain development and neural network functioning (Ingber, 2003; Jaalouk and Lammerding, 2009; Samanta et al, 2022). Notably, the composition, topography and mechanical properties of neural tissue vary between different brain regions, change during development, and contribute to the manifestation and progression of neurological diseases (Jain et al, 2020). For instance, neurodevelopmental defects linked to improper force distribution are observed in disorders such as autism, schizophrenia, and Williams syndrome. In the latter, improper force distribution could lead to cortical folding abnormalities (Franze, 2013). Taken together, this highlights the relevance of the mechanobiology properties of the neural microenvironment for brain (dys) functioning.

Since the advent of induced pluripotent stem cell (iPSC) technology, and its prospects for translational research, there has been an increase in the number of investigations leading to the development of physiologically relevant models (Vadodaria et al, 2020). iPSCs can be generated from human somatic cells and turned into any cell type of interest (Dolmetsch and Geschwind, 2011), allowing to study how human-, disease- or patient-specific genetics influence cell behaviour or result in neural abnormalities leading to specific pathological states. Mice, classically employed for these experiments, typically carry one or two genetic changes associated with a disease. However, the risk of developing a disease, the progression of a disease, and the response of a patient to drugs are influenced by a multitude of complex genetic changes or are human-specific. In addition, iPSCs also have a major advantage over human embryonic stem cells (ESCs), as ESC lines are obtained from early-stage embryos, and new line generation comes with ethical dilemmas (Rabeling and Goolam, 2022). Compared to primary cells, adult neural stem cells can be hindered by their limited capacity to proliferate and difficulties in primary isolation (Nam, 2015), while iPSCs provide an unlimited cell source for differentiation into any specialized cell type. In recent years, there has been an increasing interest in more sophisticated iPSC-based culture systems to better recapitulate the *in vivo* three-dimensional (3D) microenvironment, giving rise to brain organoids and assembloids. Compared to conventional 2D cell monolayer approaches, these 3D structures provide a more accurate representation of the brain’s *in vivo* topographical organization and cell-cell interaction. However, there is still a need for iPSC-based cultures that feature more advanced maturation and increased standardization (Andrews and Kriegstein, 2022). Considering the mechanobiological aspects during the development of new brain models, engineering approaches have prospects for improving current systems by fine-tuning the cell microenvironment (Tortorella et al, 2022). Additionally, they allow studying the biological relevance of mechanical interactions between cells and their environment to investigate healthy brain development, disease mechanisms and drug screening of treatments that target mechano-related signalling pathways (Avior et al, 2016).

The field of mechanobiology examines the influence of mechanical cues on the behaviour and phenotype of neural cells in both physiological and pathological circumstances (Lemma et al, 2019). Despite the accumulating body of evidence that illustrates the crucial role of mechanical cues in modulating neural cell behaviour (Motz et al, 2021), the mechanical aspects of the neural microenvironment are still largely ignored in current cell culture systems. Engineers and physicists have started to explore various techniques to mimic the microenvironment of cells to obtain tissue-inspired 3D cell cultures. In particular, 2D and 3D micro and nanofabrication techniques experienced rapid development and have been applied for *in vitro* cell research (Li et al, 2003). With these techniques, complex geometries can be manufactured with high resolution, and a wide range of materials can be employed. Additive manufacturing is one of the methods that can be employed to manufacture engineered cell environments. Examples of additive manufacturing techniques are two-photon polymerization (Accardo et al, 2018a; Lemma et al, 2019; Weisgrab et al, 2020; Akolawala et al, 2022; Barin et al, 2022; Sharaf et al, 2022), bioprinting (Rider et al, 2018), stereolithography apparatus (SLA, Qiu et al, 2020), and digital light processing (DLP, Qiu et al, 2020). Other techniques used to recreate microenvironments are (optical) lithography techniques (Pardo-Figuerez et al, 2018), chemical synthesis (Marcus et al, 2017), electrospinning (Honkamäki et al, 2021), salt leaching (Worthington et al, 2017), and foaming (D’Abaco et al, 2018). Various 2D, 2.5D, and 3D geometries with varying mechanical properties can be achieved by employing these techniques. Currently, the study of the mechanobiology of the brain has gained the interest of biologists, engineers, and physicists, making it now a highly multidisciplinary field (Hall et al, 2021).

This review investigates different approaches employed for mimicking the microenvironment of neural cells derived from human and rodent stem cells to develop advanced neural cell models, with a particular focus on neuro-mechanobiological properties and the different techniques to study them. Scaffold-free and scaffold-based techniques have been used to create brain models and steer neural development (Accardo et al, 2019; Valdoz et al, 2021). Scaffold-free 3D approaches employ bottom-up (*de novo*) formation and cell self-assembly to obtain multicellular 3D tissue-like cell aggregates such as spheroids and organoids (Accardo et al, 2019; Valdoz et al, 2021). On the other hand, scaffold-based techniques use biomaterials to promote the formation of 3D cell networks by providing instructive cues to the cells (Valdoz et al, 2021). The scaffold-free and scaffold-based techniques have been used with a variety of materials featuring various mechanical properties. These materials include, but are not limited to, natural ECM materials, hydrogels, and polymers (Accardo et al, 2019). The scaffolds provide a niche for the cells, support, guidance, and mechanical cues for 3D tissue formation. The following sections will discuss the fabrication and design of 2D/2.5D and 3D cell culture substrates. An overview of the advantages and limitations will be given, focusing on scaffold-based approaches. Further, we will provide an survey of the different methods for studying the mechanobiological properties of (neural) cells. This section includes a summary of the approaches used for measuring the forces that are applied by cells on their surrounding for studying mechanobiological properties. Finally, an outlook will be provided to highlight possible future strategies of 3D cultures for iPSC-derived neural cells and the possibilities of neuromechanobiological research.

## Physical properties of the brain

The brain consists of exceptionally soft tissue featuring viscoelastic properties. Furthermore, the combination of cell types, neural cell structures and extracellular matrix composition in the various regions of the brain lead to variable mechanical properties across the whole tissue (Barnes et al, 2017). This section will present an overview of the brain’s topography, stiffness and viscoelasticity as well as its relevance for neural function.

### Topography

**FIGURE 1:**
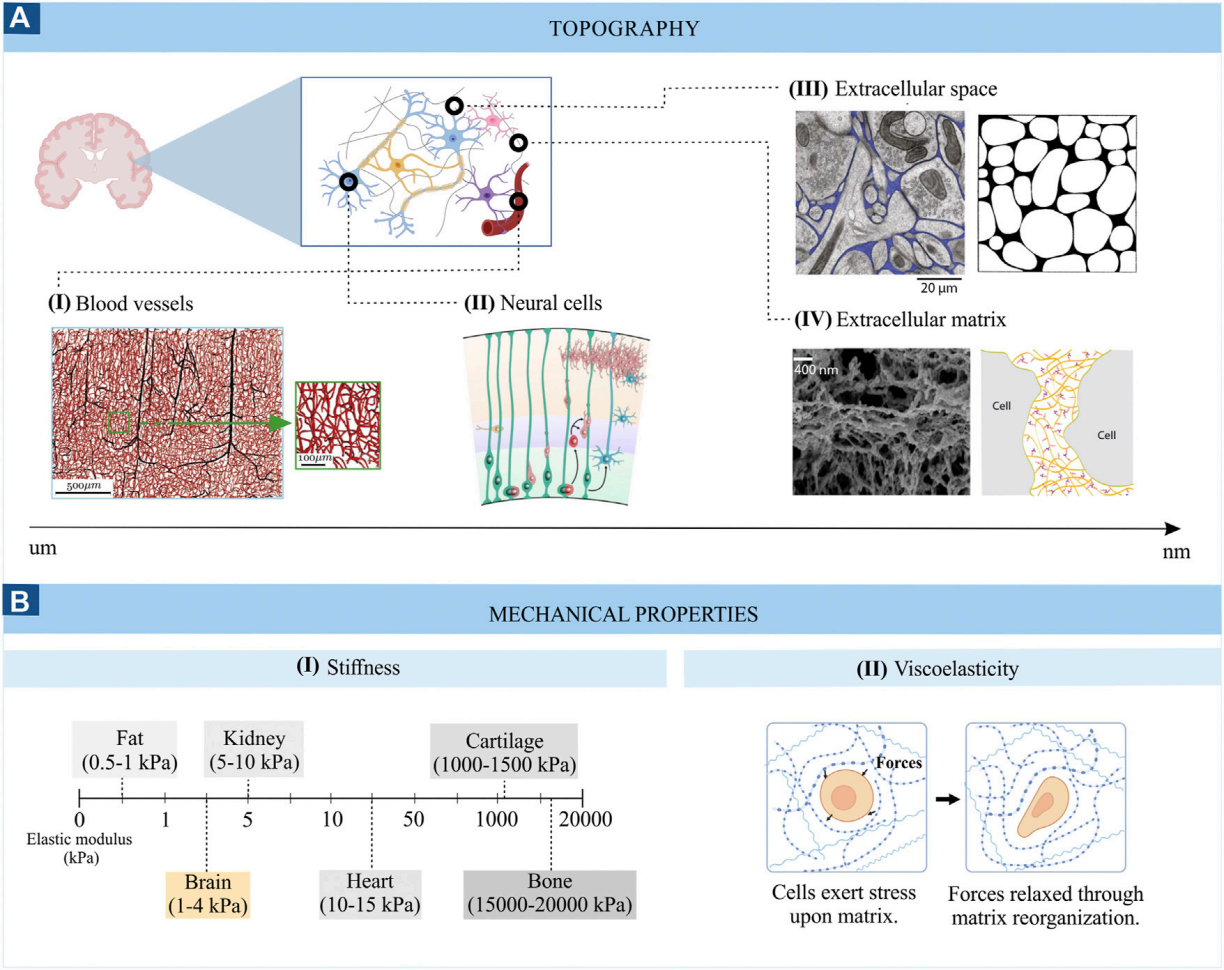
Topography **(A)** and mechanical properties **(B)** of brain tissue. Images from panel A are adapted from **(I)** Microvascular networks in the human brain (Peyrounette et al, 2018), **(II)** Schematic representation of the developing cortical plate (Barry et al, 2014), **(III)** Electron micrograph of mouse cerebral cortex where the extracellular space is coloured in blue (left) ((Korogod et al, 2015) and computer generated configurations of the brain extracellular space (right) (Chen and Nicholson, 2000); **(IV)** Scanning electron micrograph of the brain ECM (left) (Tajerian et al, 2018) and schematic representation of the ECM (right) (Odackal et al, 2017). **(B)** represents the stiffness range **(I)** and viscoelasticity **(II)** of brain tissue.

The brain topography is complex and highly organized. Different anatomic structures can be distinguished at the macroscale level, such as the brain ventricles (cavities within the parenchyma filled with cerebrospinal fluid) and the different brain regions (cerebral hemispheres, diencephalon, cerebellum and brain stem). The latter display a complex cytoarchitecture at the microscale (cellular level) leading to a broad distinction between grey and white matter. Grey matter is mainly formed by neuronal somas, accompanied by glial cells, some axons and capillary blood vessels. White matter is composed of myelinated and unmyelinated axons, along with oligodendrocytes, astrocytes and microglia cells. (Mercadante and Tadi, 2021). The topographic organization of each region is crucial for its functionality, and disruptions of this cytoarchitecture can lead to different pathologies. For example, cortical malformations may arise from alterations of the neuronal orientation and lamination (disposition on cortical layers). This change in brain cytoarchitecture can lead to pathologies such as cortical dysplasia, which usually manifests with epilepsy (Thom, 2014). Brain tissue accommodates components of different sizes and shapes, as shown in Figure 1A. The neuronal cell body, a more or less rounded structure, features approximately a diameter of 10–50 μm, while radial glial cells form fibres of 1 µm diameter. These radial glial cells serve as a scaffold for migrating neural progenitor cells during development (Cembran et al, 2020). Capillaries, veins and arteries also present a fibre-like shape, with diameters varying between 4 and 8 µm for the former and reaching a few millimetres for the latter (Mahumane et al, 2018). The capillaries, veins and arteries also serve as scaffolds for neuronal and oligodendrocyte progenitor migration (Czeisler et al, 2016). The voids between all these components form the brain’s extracellular space (Figure 1AIII), which has been described as a foam-like porous structure formed by an irregular network of highly connected cavities of various sizes and shapes (Nicholson and Syková, 1998). These voids are occupied by fluid, various solutes, and a non-cellular macromolecular framework called the extracellular matrix (ECM, Figure 1AIV). The ECM is a highly organized structure composed of several classes of macromolecules: glycosaminoglycans, proteoglycans, glycoproteins, and fibrous proteins, resembling a lattice of amorphous aggregates (Mouw et al, 2014; Roth et al, 2021). Furthermore, the ECM can be divided in different compartments with a unique composition: the basement membrane that surrounds blood vessels, the perineuronal nets that surrounds dendrites and neuronal cell bodies and the interstitial matrix that is diffusely distributed between brain cells (Kajtez et al, 2021). The ECM components present a diameter of tens to hundreds of nanometers and together form specific topographies of less than 10 μm in diameter (Jain et al, 2020). The brain ECM can thus be seen as a 3D macromolecular network that physically supports cells, fosters cell growth, maintains cell viability and has a crucial role in homeostasis and neurological diseases (Frantz et al, 2010; Rauti et al, 2020). Interestingly, the ECM composition is formed during development and is relatively stable during adulthood. For example, fibrous proteins are present in low quantities in the adult brain but are highly expressed during development (Kajtez et al, 2021). For recreating the brain ECM *in vitro*, it is crucial to take into account how specific ECM components modulate parameters such as stiffness, fibre orientation, ligand presentation and dimensionality, as they can result in specific cellular behaviour (Frantz et al, 2010). The work of Rauti et al (Rauti et al, 2020) and Frantz et al (Frantz et al, 2010) cover the chemical, structural and mechanical properties of the brain ECM and discuss the properties that *in vitro* models should be able to recapitulate. The reader is referred to their works which describe the methodologies and challenges involved in creating a synthetic brain ECM in more detail.

Another relevant topographical feature of the brain tissue is its structural anisotropic nature. This anisotropy is one of its most distinctive features and originates from the alignment of neuronal axons in the white matter. This directionality does not prevail in the grey matter, which contains relatively few myelinated axons and is mainly occupied by cell bodies (Ayad et al, 2019).

### Stiffness or elasticity

The stiffness of the brain, measured by the Young’s modulus (also called the elastic modulus, i.e. the resistance of the material to elastic deformation), is quite heterogeneous, ranging between 0.1 and 1 kPa in healthy brain tissue (Figure 1BI) and reaching values of 10–16 kPa in diseased states (Bizanti et al, 2021). This low stiffness results in part from the specific composition of the brain extracellular matrix, which is mainly made up of glycosaminoglycans (e.g., hyaluronic acid), proteoglycans (e.g., aggrecan), glycoproteins (e.g., tenascin-R) and low levels of fibrous proteins (e.g., collagen and fibronectin). The lack of fibrous proteins is responsible for the low stiffness of the brain compared to other tissues (Motz et al, 2021). Comparing different neural cell types, glial cells are generally less stiff than neurons (Lu et al, 2006). Interestingly, a closer look at different neuronal cell types reveals varying stiffness. For example, cortical neurons are softer than hippocampal neurons (30–500 Pa *vs*. 480–970 Pa) (Procès et al, 2022). Similarly, different stiffness values can be found among glial cells and brain regions. For example, white-matter-derived microglia and astrocytes are softer than their grey-matter-derived counterparts (842 *vs*. 1439 Pa for microglial cells and 1.5 *vs*. 2.7 kPa for astrocytes) (Antonovaite et al, 2020; van Wageningen et al, 2021). The importance of tissue stiffness is especially clear during brain development when stiffness gradients guide axon growth, and unusual tissue stiffness can lead to aberrant axon growth (Koser et al, 2016). The stiffness can thus play a crucial role in disease states as well, which is exemplified by brain tumours, where the progression is related to an increase in the stiffness of the tumour-associated ECM (Ayad et al, 2019). Thus, the cellular composition of brain networks and their extracellular matrix are distinct for different brain regions and is influenced by age, developmental state, and pathological state. Measuring the elastic modulus allows to monitor these changes closely related to cellular function.

### Viscosity

Brain tissue also displays viscous characteristics (Figure 1BII), which, together with its elastic properties, confers the viscoelastic behaviour to the brain. Viscoelastic properties allow brain tissue to withstand deformation upon applied forces by dissipating the forced-derived energy and reorganizing its structure (Kratochvil et al, 2019). In other words, in response to a mechanical perturbation, like compression, the brain tissue first deforms (elastic response) and then dissipates part of this energy in a process called “stress-relaxation”. For example, the lipid bilayer that forms the membrane of neurons and glial cells can deform and regain its shape in response to physical forces, allowing cells to extend and retract protrusions necessary for cell migration or withstand the forces generated during a traumatic brain injury (Tyler, 2018). Similarly, the brain’s extracellular matrix can rearrange its structure after being deformed by forces imposed by neural cells (Procès et al, 2022). Differences in the neural tissue’s viscoelastic properties are also found throughout the brain. For example, white matter is, in general, more viscous and exhibits longer relaxation times compared to grey matter (stress relaxation times of white matter ≥600s *vs*. grey matter = 400s) (Budday et al, 2015). Since the plasma membrane of neuronal and glial cells also displays viscoelastic features, this property is also observable at the microscale. Interestingly, glial cells are more viscous than neuronal cells, suggesting they could provide a compliant embedding for neurons (Lu et al, 2006). Furthermore, differences in the viscoelastic properties of grey and white matter microglia have been found in response to an inflammatory stimulus (van Wageningen et al, 2021), highlighting the role of brain viscoelasticity in neuropathology as well. The viscoelasticity of brain tissue also affects brain function at many other levels (Tyler, 2018). For example, the viscoelasticity of the neuronal plasma membrane can modulate the opening of stretched-activated ion channels, influencing neuronal excitability (Tyler, 2018). In conclusion, brain tissue mechanics are crucial to brain function and can contribute to the emergence and development of neurological diseases. Therefore, introducing these parameters in current *in vitro* brain models is essential to recapitulate the *in vivo* neural environments and investigate cell-environment interactions, which will be examined in the following section.

## Cell-environment interactions: Mechanotransduction

**FIGURE 2:**
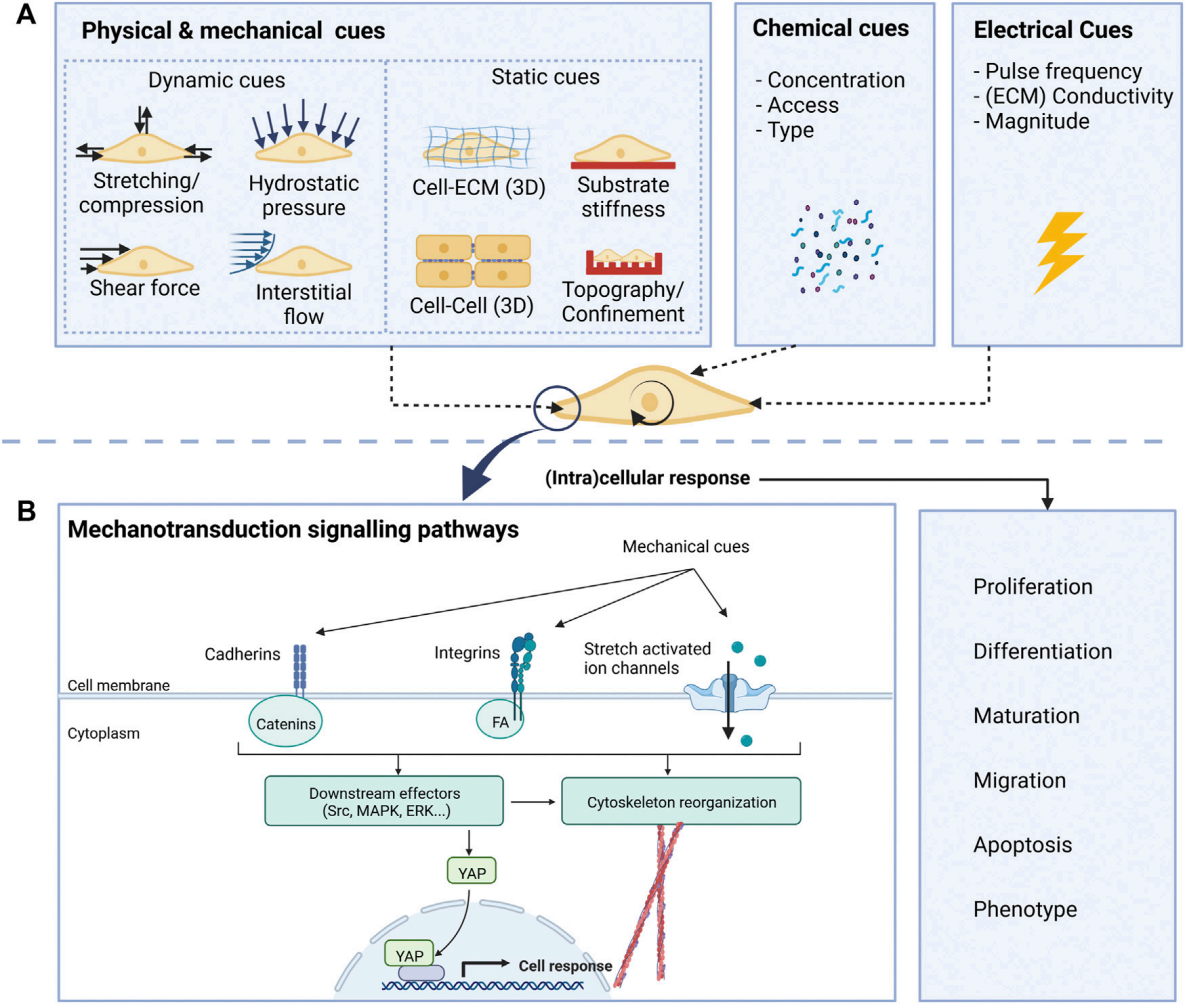
Overview of relevant cues **(A)** for neural cells and mechanotransduction pathways **(B)**.

Along with the mechanical and topographical cues mentioned in the previous section, the neural environment presents other mechanical cues to neural cells, including physical forces exerted by the neighbouring cells, compression loading, and shear stress caused by cerebrospinal fluid flow (Gargalionis et al, 2022). The process through which cells integrate these mechanical cues is called mechanotransduction and is mediated by specific intramembrane protein structures called mechanosensors (Gargalionis et al, 2022). These, in turn, transduce the stimuli to intracellular adaptor proteins, which can interact with downstream intracellular and nuclear signalling molecules or the actomyosin cytoskeleton. In the end, these signalling cascades lead to cell morphology and gene expression changes, modulating cell behaviour (Marinval and Chew, 2021). A simplified scheme of these mechanisms is depicted in Figure 2B.

Integrin proteins are the main mechanosensors that mediate cell-substrate interactions and are essential for axonal pathfinding and dendritic spine and synapse formation (Kajtez et al, 2021). Upon their stimulation, integrins activate adaptor proteins, such as those of the focal adhesion complex (FA): vinculin, talin, paxillin, and focal adhesion kinase (FAK). Interestingly, the recruitment of these proteins to the FA is majorly regulated by the forces exerted on the integrins. For example, vinculin recruitment is favoured by applying tensile forces (Stukel and Willits, 2016). Proteins from the FA link the *β* subunit of integrins to the actin cytoskeleton, leading to dynamic modifications of the cytoskeleton organization and tension (Eyckmans et al, 2011). They also trigger numerous intracellular signalling cascades upon phosphorylation of FAK, such as the Rho/ROCK and the ERK1/2 signalling pathways, in addition to other cascades involving proteins like Src family kinases, Rac or Cdc42, which are involved in numerous cell functions including migration, differentiation and proliferation (Stukel & Willits, 2016). Cell-substrate interactions can also be mediated by stretch-activated ion channels, like Piezo-1. These channels can detect mechanical forces and displacements and allow the entry of ions into the cell, activating a series of downstream signalling pathways that will modify cell behaviour (Rocha et al, 2022). Two examples are Piezo 1 and Piezo 2 cation channels that can open in response to mechanical forces. These cues can be generated either inside the cell, as changes in cell membrane tension, or externally, due to variations of the microenvironment stiffness (Hall et al, 2021). Interestingly, Piezo1 has been proven to mediate lineage specification of neural stem cells (Pathak et al, 2014) and axonal growth patterns (Motz et al, 2021) in response to substrate stiffness. Furthermore, Piezo channels allow neurons to sense astrocyte stiffness, thereby mediating neuron-astrocyte interactions (Motz et al, 2021). Cells also interact with other neighbouring cells and do so mainly through cadherins. These mechanosensory proteins interact with several adaptor molecules, typically proteins from the catenin family. As before, adaptor proteins bind to the cytoskeleton, which is linked to the nuclear membrane, eventually modifying gene expression. The cytoskeleton is essential in mediating force transmission and constitutes a dynamic structure that can modify its organization, allowing cells to adapt and respond to their surroundings and connect with other mechanosensing structures (Eyckmans et al, 2011). Also important are nuclear mechanotransducers, such as YAP (Yes-associated protein). YAP and its homolog TAZ are key mediators of the effects of mechanical stimuli on cell behaviour (Motz et al, 2021). A better understanding of how neural cells interact with their environment, the molecular players and signalling pathways that translate mechanical inputs into cell behaviour is key to designing engineered culture systems that can steer cell phenotype in the desired direction (e.g., neuronal differentiation, increased myelination, etc.).

## Mimicking the brain microenvironment *in vitro*

Mimicking the natural microenvironment in cell culture systems makes models more physiologically relevant. Therefore, providing biochemical and geometrical cues comparable to the natural environment is essential for realistic tissue development and cell behaviour. During the past two decades, researchers aimed at the optimal synergy between engineered scaffolds and cells by testing different biomechanical, biochemical and biophysical cues that support the cell’s maintenance, function, proliferation, and differentiation (Batorsky et al, 2005; Bambole and Yakhmi, 2016).

### Current *in vitro* models

Most of the current cell research is being performed using 2D culture platforms. These are easy to handle, inexpensive, and offer better accessibility for observation and manipulation. In addition, they offer higher reproducibility and are well suited for simple comparative functional tests (Duval et al, 2017; Kapałczyńska et al, 2018), often leading to relatively homogeneous cultures with few cell types (Sun et al, 2018). Conversely, 3D models better resemble cellular heterogeneity, spatial organization and temporal development of neural tissue (Kajtez et al, 2021). Nevertheless, they are hampered by reduced reproducibility, extensive culture times and complicated analysis and manipulation (Pampaloni et al, 2007). Table 1 presents an overview of the advantages and disadvantages of 2D and 3D culture systems.

**TABLE 1:** Overview of 2D *vs*. 3D *in vitro* brain models with related advantages (+) and disadvantages (-) (Baker and Chen, 2012; Kapałczyńska et al, 2018; Sun et al, 2018).

	2D neural cell culture 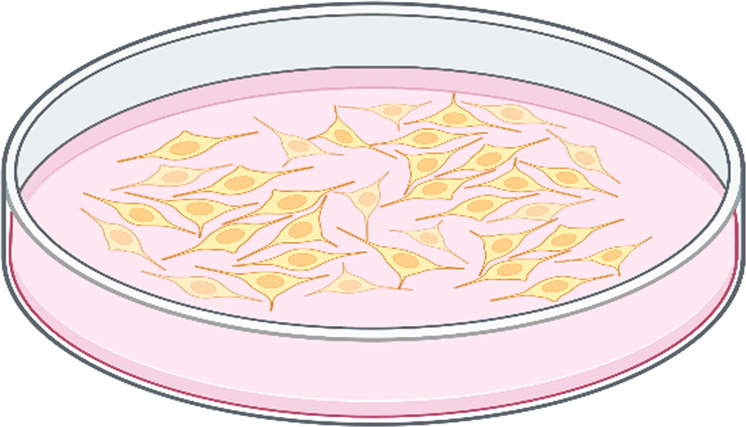	3D neural cell culture 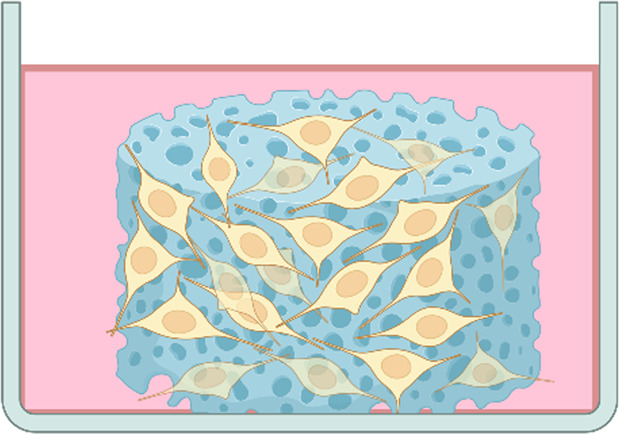
*In vivo* imitation	- Cannot recapitulate 3D natural structure or organization	+ Can mimic 3D natural structure or organization
	- No in vivo-like 3D microenvironment	+ In vivo-like 3D microenvironment possible
	- No niche formation	+ Niche formation
	- Simple 2D neural circuits can be investigated	+ Complex 3D neural network formation
	- Altered morphology	+ *In vivo* morphology maintained
	- Changes in gene expression	+ Similar gene expression as *in vivo*
	- Loss of phenotype and polarity	+ Diverse phenotype and polarity
	- Functional maturity after long cultures	+ Enhanced functional maturity
	- Unconstrained spreading and migration in x-y	+ Spreading and migration partially hindered and in 3D
	- Soluble gradients absent	+ Soluble gradients can be present (e.g. organoids and gel cultures)
Cues	- Simple and limited cell-cell and cell-ECM interaction, reduced to 2D	+ Proper cell-cell and cell-ECM interaction possible in 3D
	- Limited Physical and mechanical cues reduced to 2D	+ Physical and mechanical cues possible in 3D
	- Only 2D mechanotransduction	+ Realistic 3D mechanotransduction
Culture	+ Fast (culture formation within a few minutes to a few hours)	- Slow (culture formation within a few hours to a few days)
	+ Reproducible	- Lower reproducibility
	+ High throughput possible	- Low throughput compared to 2D cultures
	+ Cheap	- Expensive
	+ Less laborious	- Laborious
	+ Simple culture steps, analysis and interpretation	- Complex culture steps, analysis and interpretation
	+ Simple cell isolation	- More complex cell isolation and smaller yield in scaffold-based cultures

Most of our knowledge of mechanobiological interactions between neural cells and engineered microenvironments comes from studies of cells cultured on 2D platforms. These 2D platforms help us understand basic cellular interactions with a material but do not resemble the 3D environment cells encounter *in vivo* (Stukel and Willits, 2016). Most importantly, there are differences in how mechanical and topographical cues are sensed by cells in 2D and 3D environments (Baker and Chen, 2012). Cells cultured on 2D substrates can deform in the out-of-plane axis, perpendicular to the surface, and are free of physical or mechanical constrains. In contrast, cells may be constrained by 3D constructs, which influence cell deformation and how forces are transmitted to cells, depending on the material’s scale and mechanical properties (Baker and Chen, 2012).

Cell-material interactions also differ at the integrin level in 2D and 3D cultures. In 2D cultures, adhesions to the substrate are formed only on one side of the cell (x-y plane), leading to a force transmission along the cytoskeleton stress fibres located on that side of the cell. In 3D cultures, these adhesions can be found all over the cell’s surface, causing forces to convey along the cell’s midline. This differential spatial distribution of adhesions could lead to different cellular responses (Baker and Chen, 2012). 2D platforms also lead to a flattened and altered morphology of cells, which restricts cell-cell interactions mainly to side-by-side contact with consequent relevant modifications of gene expression and network activity (Kajtez et al, 2021). Furthermore, forces exerted by cells on their surroundings have different results in 2D and 3D settings, as the adhesion in 2D cultures is restricted in the x-y plane, while in 3D, the adhesion is distributed in all directions (Baker and Chen, 2012). Thereby, traditional 2D culture settings can provide basic information regarding how cells sense and respond to different mechanical cues. However, the differences in cell behaviour in 2D and 3D environments and the fact that the latter grants a more physiologically relevant representation of the geometrical and mechanical features of the *in vivo* cell niche emphasize the need to investigate how our current understanding of mechanobiology translates to a 3D settings.

### Scaffold-free *vs*. scaffold-based 3D cell cultures

3D cell culture techniques can be divided into scaffold-free and scaffold-based techniques. Scaffold-free techniques (also referred to as “anchorage-independent technologies”) are based on *de novo* formation of 3D cell clusters through self-aggregation (Langhans, 2018; Accardo et al, 2019; Valdoz et al, 2021). Special culture plates/techniques are used for scaffold-free cultures, such as hanging drop microplates, ultra-low adhesion plates, micropatterned plates and magnetic levitation. These plates promote the formation of spheroids and organoids (Langhans, 2018). The formed cell clusters can capture the cell-cell interaction between different cell types and have been used to perform drug screening tests (Langhans, 2018). Additionally, they resemble the cells’ physiological conditions much better than the monolayers regarding both the nutrient supply and the spatially defined differentiation. However, culturing these organoids requires extensive optimization of the culture conditions and has a relatively low reproducibility. In addition, when the cell cluster becomes too large, the nutrient supply can become insufficient, resulting in an early necrotic core, which is detrimental, especially in view of long-term (weeks/months) cultures (Grebenyuk and Ranga, 2019).

Scaffold-based culture techniques use (simple) mechanical structures to physically support and guide 3D neural cell growth and network formation. The structures provide mechanical support and form a matrix into and onto which the cells are cultured. As the cells receive cues from the microenvironment, the physical and chemical properties of the scaffold will influence the cell’s behaviour, differentiation, migration and proliferation. Researchers aim to create 3D scaffolds from polymeric biomaterials that are analogous to the natural ECM (Bambole and Yakhmi, 2016). The scaffolds can be biologically active and provide structural and physical support to cells by serving as a matrix, which allows cells to adhere, proliferate and differentiate, enabling neo-tissue genesis and natural ECM deposition (Bambole and Yakhmi, 2016). Engineered scaffolds often require a coating, such as a protein or peptides, to improve cell interactions (e.g. cell attachment) and matrix degradation (Bertucci and Dai, 2018).

### Incorporating physical cues within *in vitro* models

*In vivo* cell migration, proliferation and differentiation are continuously influenced by environmental cues, which must be considered when replicating the environment for *in vitro* studies. The cues can be split into two groups: static cues and dynamic cues. Figure 2 schematically shows the cues acting on cells *in vitro*. By considering both the static and dynamic cues during the development of cell culture environments, it is possible to create highly biomimetic microenvironments.

#### Static cues

The engineered microenvironments’ static cues (Figure 2A) can be designed and tuned during fabrication. These cues include substrate stiffness, surface topography, and the (3D) geometry. The stiffness of the substrate can influence the differentiation of iPSCs towards different lineages (Macrí-Pellizzeri et al, 2015). In addition, substrate stiffness affects cell spreading and the shape of a cell, with higher stiffness generally promoting cell spreading and lower stiffness leading to more rounded cells (Knothe Tate et al, 2008; McBride and Knothe Tate, 2008; Petzold and Gentleman, 2021). Stiffer substrates generally increase the tension in the cell as the cells cannot deform the matrix. The cells react to the internal tension by spreading over the substrate (McBride and Knothe Tate, 2008). With soft substrates, the cells can deform it and thus do not need to generate large tension forces. Therefore, they can retain their round shape (Knothe Tate et al, 2008; Petzold and Gentleman, 2021). Selecting tissue-specific properties is, therefore, essential in choosing the matrix stiffness for cell cultures that mimic the *in vivo* environment, resulting in a more realistic cell phenotype and cytoskeletal organization. For neural studies, this means that a stiffness of 0.5 kPa would be optimal, as it resembles the stiffness of the brain tissue (Rehfeldt et al, 2007). However, the surface topography can alter the cell’s perceived stiffness of its substrate. For example, cells cultured on high aspect-ratio and bendable pillars behave similarly to those on soft ECMs (Nikkhah et al, 2012; Petzold and Gentleman, 2021; Sharaf et al, 2022). The interaction between cells but also between cells and their environment *in vitro* can alter the cell phenotype and ECM structure (Holle et al, 2018). For example, when a cell binds to an ECM fibre, it transmits a force to the ECM fibre. The matrix can be rearranged by the cells binding to it and pulling on it. Though, the resulting tension in the fibre can also be sensed by another cell at a distance, which shows that the cell-ECM interaction can also influence cell-cell communication (Holle et al, 2018).

The cells and the components of the ECM can also impose confinement on the cells, which occurs both *in vitro* and *in vivo*. The cells respond to the physical constraints by altering their migration, signalling pathways, intracellular cytoskeleton, and adhesion organization (Paul et al, 2016). The physical confinement typically causes changes in the cytoskeletal architecture, such as the alignment of cytoskeletal components in the direction of cell migration. If a microchannel confines a cell, actin can accumulate around the cell cortex, and stress fibres can be suppressed (Paul et al, 2016). Studying the effect of confinement on cell migration in 2D does not recapitulate the complex topographies found in the body. Recapitulation of the key static cues of the *in vivo* microenvironment is, therefore, essential to obtain physiological-relevant results.

#### Dynamic cues

In contrast to the static cues, dynamic cues (Figure 2A) can be altered during the cell culture. These cues include fluid flow, (hydro) static pressure and forces on the cell (compression and tension), and can influence the cell’s fate, mobility, behaviour, and shape. It was shown that in response to fluid flow, electrical stimulation and culturing on scaffolds, the mechanotransduction pathway gets activated, modulating changes in the cytoskeletal tension and gene activity and subsequently leading to increased differentiation of neural cells (Grossemy et al, 2021).

Several other studies investigated the effects of dynamic cues on cell maturation *in vitro*, even though there are limited biophysical tools available. For example, by applying stretching cues (different stretching modes) combined with micropatterning and microfluidics, neural stem cells showed increased differentiation towards neurons with enhanced neurite extension, axon elongation, and neurite alignment (Chang et al, 2013). Arulmoli et al (Arulmoli et al, 2015) found that statically stressed membranes increased the differentiation of neural stem cells and neural progenitor cells into oligodendrocytes. Magnetic manipulation, another technique to investigate the effect of stretching and resulting nano-pulling, promoted neural differentiation, axonal elongation, sprouting, and neuron maturation (Vincentiis et al, 2022). In addition, Vincentiis et al (Vincentiis et al, 2022) showed that the nano-pulling stimulation led to a reorganization of the neural network and remodelling at the level of synapse density, halving the required time for maturation of neural precursors into neurons.

Recent bioengineered microfluidic organ-on-chip-based models investigated the effects of flow-induced shear stress on iPSCs (Workman and Svendsen, 2020). Here the focus has been on the blood-brain barrier function, as flow-induced shear stress can promote ESC differentiation towards hematopoietic and endothelial cells. In combination with surface patterning, it was shown that flow-induced shear stress also improved neuronal differentiation (Jeon et al, 2014). Riehl et al (Riehl et al, 2015) showed that fluid shear promoted neural cell migration in the direction of the flow and that this was proportional to the stress. Their results indicate that focal adhesion kinase (FAK) and RhoA kinase (ROCK) play an important role in this process. Kim et al (Kim et al, 2006) combined mechanical stimuli with micropatterned substrates by employing a flow chamber to apply shear stress on micropatterned substrates. This technique could apply continuous or intermittent shear stress on the cells. They found that a shear stress of 0.5 Pa resulted in a high degree of alignment with the microfibers, while the neurite outgrowth was largest at a shear stress of 0.25 Pa.

The aforementioned cues do not only affect neural development at the cell level. Organoids are also affected by cues from their environment. Karzbrun et al (Karzbrun et al, 2018) showed that folding and wrinkling of a lissencephalic organoid occurred as a result of the strain caused by compression. They captured the physics behind the folding phenomenon, indicating that cues should be considered at the multiscale. In summary, the development of new engineered scaffolds and chip technologies will help to explore the role of dynamic cues in advancing brain model systems.

## Fabrication techniques and materials for scaffold-based 3D cell culture

**FIGURE 3:**
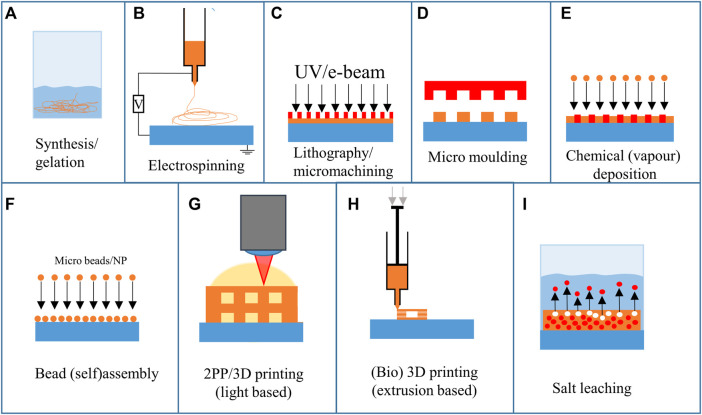
Overview of fabrication techniques employed for the realization of neural scaffolds.

Ideally, 3D scaffolds for cell culture are biocompatible and have mechanical properties similar to the specific type of natural tissue (Bambole and Yakhmi, 2016). Several fabrication techniques have been employed to satisfy these requirements. Figure 3 shows the large variety of approaches employed to fabricate scaffolds for 2.5D and 3D cell cultures. The materials that can be used differ for each technique. In addition, some techniques have full control over the feature geometry, others have limited feature resolution (tens of micrometres), while others enable the fabrication of very complex and accurate geometries (submicrometric feature size). The detailed explanation of the working principle of these techniques goes beyond the scope of this review, but the basic mechanisms of the techniques that have been employed for neural cultures will be summarized.

Chemical synthesis and gelation employ chemical reactions or the formation of a gel from a system containing monomers and polymers, respectively (Figure 3A). However, these techniques have no to poor control over the spatial or geometrical configuration of the final structure.

Electrospinning is an often-used technique to create micro and nano fibres for 3D cell culture (Figure 3B). Electrospinning uses high electric voltages (exceeding tens of kilovolts) to create a potential difference between a spinneret and a collector (Smith and Mele, 2021). This results in an electrified polymer jetting from the tip of the spinneret. As the polymer stretches while jetting, the fibre’s diameter decreases, resulting in ultra-thin fibres. By altering the setup, the fibres can be randomly deposited, have a high degree of alignment, and have varying porosity and chemical composition. Electrospinning has also been combined with 3D printing to create multiscale composite scaffolds (Smith and Mele, 2021). The fibres can either have random directions or be aligned in a specific direction.

Lithography and micromachining techniques (Figure 3C) have been used to create 2.5D scaffolds, patterns and pillars. To create the structures, a pattern is transferred from a mask to a photosensitive polymer layer (Hines et al, 2011). According to the nature of the photopolymer (negative or positive tone), it is possible to obtain either polymeric 2.5D scaffolds (Accardo et al, 2018b) or silicon ones (Limongi et al, 2013). Repeating this 2D process allows various structures to be fabricated in 2.5D or 3D with limited design freedom. The lithography and micromachining steps are often combined with micro moulding (Figure 3D), which was recently employed for neural implants (Vaysse et al, 2015). The negative mould, which is used to transfer a pattern onto a surface for micro moulding, is often fabricated using micromachining techniques. Next to lithographic methods, replication methods and material removal methods can be used to create the moulds (Yao, 2009). Additive manufacturing techniques have also been used to create moulds that can then be used to fabricate larger batches by replication. The main limitation of micro moulding is that the mould must be released after stamping. This limits the 3D design freedom considerably.

Chemical (vapour) deposition (CVD, Figure 3E) uses reactive species that are deposited on a surface. By using masks and templates, specific areas can be coated with the desired material. Depending on this material, solid and porous structures can be created. As the technique is multidirectional, the design freedom is limited. However, by combining CVD with a template, 3D structures can be created (template-directed chemical vapour deposition). Chen et al (Chen et al, 2011) employed this method to create graphene foams, which are flexible and electrically conductive. D’Abaco et al employed similar graphene foams to culture neural cells (derived from ESCs) and proved that they maintain their viability and that neuronal differentiation is similar to 2D cultures (D’Abaco et al, 2018).

The bead (self) assembly principle (Figure 3F) employs particles (with sizes ranging from several nanometres to hundreds of micrometres in diameter) that assemble on a surface. The self-assembly process relies on the spontaneous organization of microbeads on a surface. By rubbing the beads in a specific direction, an organized pattern of microbeads is formed on a surface (Kang et al, 2014).

3D printing (Figures 3G, H) experienced a huge increase in interest over the past decades. Both light-based and extrusion-based techniques have been employed to create substrates and niches for cells. 3D printing allows for a high freedom of design and fast iterations at low cost. The 3D printing technique with the highest freedom and feature resolution is two-photon polymerization (2 PP). This technique employs non-linear photon absorption to cure a photoresist and create a 3D geometry by scanning through the resin. To achieve this high resolution, a near-infrared (NIR) femtosecond laser source is used. At the location where two photons are absorbed, a small volume (voxel) of photosensitive resist is polymerized. By scanning the voxel through the photosensitive resist, a 3D structure can be obtained. Depending on the methods and materials used, features can be achieved with 100 nm resolution (Qiu et al, 2020). The high resolution provides a good level of reproducibility and facilitates the reliability of cell culture experiments. Koroleva et al employed 2 PP printing to culture neural cells from iPSCs (Koroleva et al, 2021), which is discussed in more detail in *3D Architectures Section*.

Another technique that has been used to create 3D microenvironments is bioprinting (Figure 3H). This method creates 3D structures consisting of biomaterials integrating cells, and biomolecules (Rider et al, 2018). Bioprinting can be based on various additive manufacturing techniques such as extrusion-, inkjet-, and optical-based systems (Rider et al, 2018). These techniques enable the printing of gels at low temperatures, but have a limited resolution (resolution of tens to hundreds of micrometres). Both bioprinting (Sharma et al, 2020) and 2 PP printing can be performed with (iPSC) cells embedded in the resin (Tromayer et al, 2017, 2018; Dobos et al, 2020). Bioprinting iPSCs is challenging as they are sensitive to environmental parameters (Koch et al, 2018). Koch et al showed that iPSC cells died in some printing materials regularly used for printing other cell types. However, they demonstrated that laser bioprinting of iPSCs is possible and that the cells maintain their differentiation potential. An extensive review of 3D printing techniques for engineered polymer and hydrogel microenvironments has been performed by Fan et al (Fan et al, 2019).

Salt leaching (Figure 3I) can be used to fabricate random porous scaffolds. One of the possible configurations for this technique employs a polymer mixed with salt crystals. After melting the polymer mixed with salt into a mould, the salt can be removed by submerging it in deionised water. The water then leaches out the salt, leaving a porous geometry (Cho et al, 2014). The limiting factor is the random distribution of salt crystals. This results in a random distribution of pores, which can be favourable depending on the type of research. However, it can affect the reproducibility of the experiment.

## Techniques to measure cell mechanical properties

**FIGURE 4:**
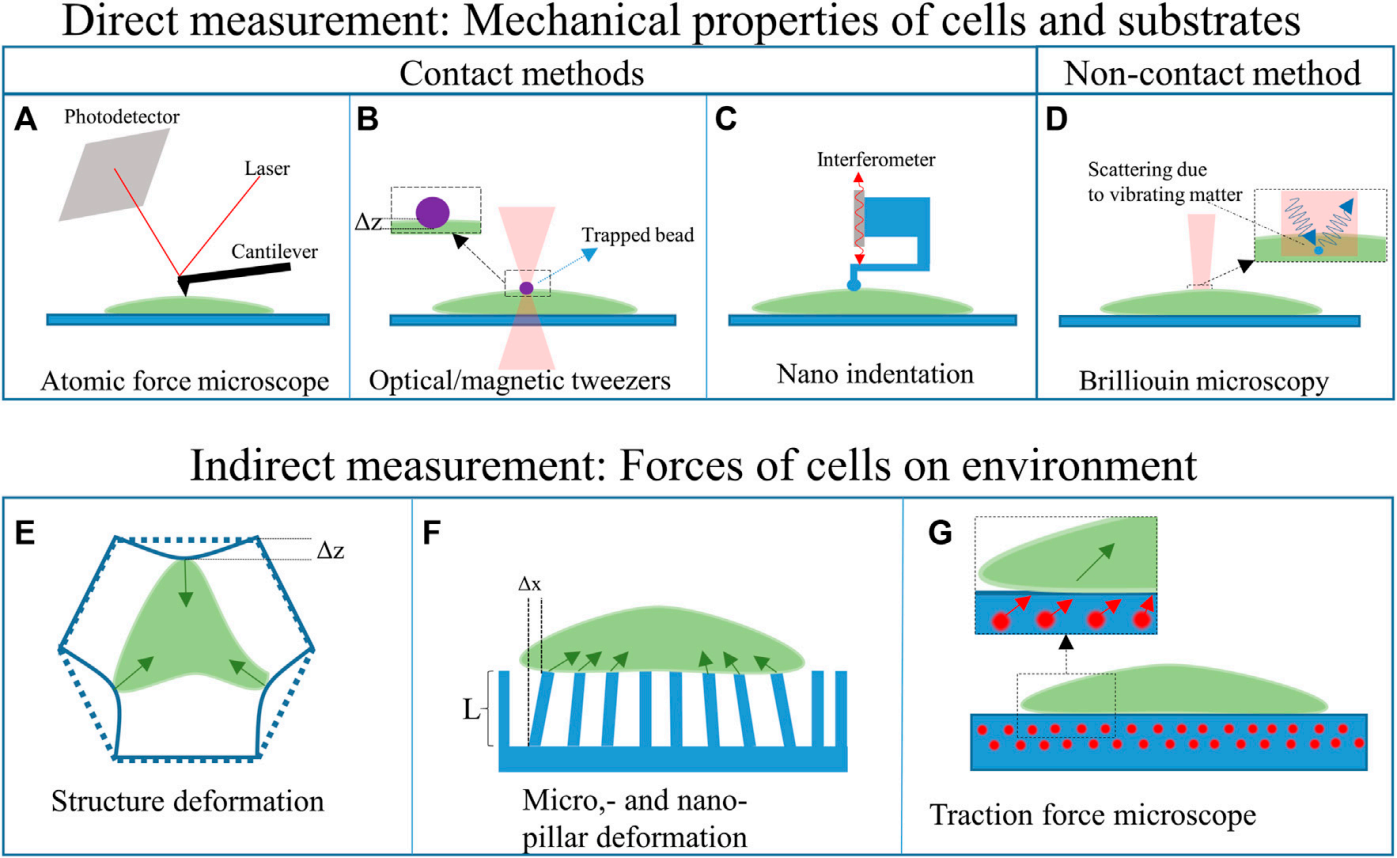
Overview of the techniques employed to measure cell mechanical properties.

Besides fabricating biomimetic scaffolds to foster the creation of physiologically relevant neural networks, it is also important to develop approaches enabling the measurement of cellular forces, thus unveiling neuromechanobiology properties. The mechanical properties of cells can be studied in 2D and 3D cell culture platforms. Figure 4 shows various techniques that can be applied for studying the mechanical properties of cells and their response to different material properties of the substrate or (artificial) ECM. Two main groups can be distinguished: direct and indirect mechanical property measurements. The direct measurement techniques probe the cell itself to determine its mechanical properties. The indirect measurement techniques, on the other hand, use the deformation of the substrate or a structure, caused by the cell, to determine its mechanical properties. These techniques can be used to determine the response of the cell to various cues, such as substrate stiffness.

### Direct mechanical property measurements

Among the direct measurement techniques, another subdivision can be made, namely: contact- and non-contact-based methods. The contact-based methods, such as atomic force microscopy, optical and magnetic tweezers, and nanoindentation, apply forces on the cell. The forces, in combination with the deformation and geometry of the device that indents/interacts the cell, can be used to calculate its mechanical properties of cells. The non-contact-based method, such as Brillouin microscopy, does not apply forces on the cell. All these techniques can be used to characterize both the cells and the substrates (Narasimhan et al, 2020).

#### Contact based methods

Atomic force microscopy (AFM) employs the deflection of a sharp-tipped cantilever to probe the mechanical properties of a cell (Figure 4A). The cantilever tip scans the sample’s surface to extract 3D topographic information and Young’s moduli of the surface (Jazvinšcak Jembrek et al, 2015). AFM techniques have also been combined with hollow microfluidic cantilevers called FluidFM (Meister et al, 2009), which can, for example, be employed to determine the adhesion forces between a cell and the substrate. AFM techniques have been used to study the nanostructural, morphology and biomechanical properties of neurons (Jazvinšcak Jembrek et al, 2015), such as nanoscopic changes in the plasma membrane caused by oxidative damage. This oxidative damage is a hallmark of some neurodegenerative diseases (Uttara et al, 2009; Jazvinšcak Jembrek et al, 2015). AFM measurements have also expanded our understanding of the processes occurring in neurodegenerative diseases and showed the (mechanical) response of neurons to different drugs and neurotoxins (Jazvinšcak Jembrek et al, 2015). Additionally, AFM has been used to correlate the elastic behaviour to cell migration and division (Jazvinšcak Jembrek et al, 2015).

Two other methods that can be used to determine the mechanical properties of neurons are optical and magnetic tweezers (Chen et al, 2016; Lenton et al, 2020). Optical tweezers (Figure 4B) use light to trap micrometric particles. The particles become trapped at the beam focus if the optical forces are larger than the other acting forces. This technique has been used to trap silica beads to deform glial cell surfaces and measure their stiffness and elasticity (Dagro et al, 2019). The main advantage of this technique is that it can be used in a 3D scaffold environment and measure forces in a 3D space (Lenton et al, 2020). However, forces that can be applied are limited to the piconewton range (Marti and Hübner, 2010).

Magnetic tweezers apply a similar concept as optical tweezers but use a gradient of the magnetic field to trap a magnetic bead (Chen et al, 2016; Sarkar and Rybenkov, 2016). The bead can then be used to indent a neural cell and determine the mechanical properties of the cells (Chen et al, 2016). Magnetic tweezers track the position of magnetic beads in a 3D space and perform better in the presence of a homogeneous field (Sarkar and Rybenkov, 2016).

Nanoindentation (Figure 4C) can be performed using either a force sensor or a cantilever linked to an interferometer. The former can employ micro-electro-mechanical systems (MEMS) technology to fabricate highly sensitive load cells (measuring the capacitance change of interdigitated electrodes) to measure the forces during nano-indentation (e.g. Femtotools, Switzerland). The interferometer-based system, such as the Piuma (Optics11, Netherlands), measures the deflection of a cantilever with a spherical glass tip. If the cantilever deflects, the interference pattern changes. The measured deflection, in combination with the known displacement of the probe and the characteristics of the cantilever and tip, can then be used to calculate the Young’s modulus of a material. The indentation depth of this system can range from hundreds of nanometres to several micrometres. The main difference with AFM systems is that an AFM is based on the tip-surface interactions and has a much higher lateral resolution (can measure in the piconewton range). Next to measuring the Young’s modulus, it can also map the surface topography. However, the nanoindenter setups typically require less sample preparation, less alignment, can apply larger forces and can provide fast measurements (Kong et al, 2021).

#### Non-contact-based methods

Non-contact-based methods do not use any solid parts that come into contact with the cells. An example is (confocal) Brillouin optical microscopy (see Figure 4D). Brillouin microscopy employs Brillouin scattering of a monochromatic laser light arising from the interaction of light with spontaneous, thermally induced density fluctuations in a cell (Prevedel et al, 2019; Zhang and Scarcelli, 2021). The interaction between the incident laser light and the acoustic waves introduces a frequency shift (Brillouin shift) to the scattered radiation due to the Doppler effect (Prevedel et al, 2019). As the propagation of the acoustic wave is dependent on the properties of the material, it is possible to deduce these properties by analysing the frequency shift (Prevedel et al, 2019; Zhang and Scarcelli, 2021).

### Indirect force measurements

The indirect force measurement techniques are used to characterize the cell-structure interaction (Narasimhan et al, 2020). Figure 4 shows three different methods used to determine the interaction between the cells, the substrate or the surroundings. All these techniques measure the deformation of the environment, with a known stiffness, for determining the properties of the cell.

Structure deformation of 3D scaffolds has been used to determine the interaction of cells with elastic 3D structures (Klein et al, 2010). An illustration of this principle is shown in Figure 4E. By printing structures with beams featuring a known cross-section and Young’s modulus, the forces of the cell on the structure can be determined measuring the deflection of the beams. The latter can be quantified by employing analytical and numerical tools. The structures of Klein et al were printed using two-photon polymerization, which allows for the fabrication of accurate beam sizes (Klein et al, 2010). The deformation of the structure can be measured in a stressed state (with cells) and compared to the relaxed state (without cells) either with optical tools (e.g. confocal microscope) or by using a scanning electron microscope (SEM). To measure the deflection of the substrate, a comparison needs to be made between the relaxed state and the stressed state (without and with cells, respectively).

Similarly, pillar deformation (see Figure 4F) employs the deformation of pillars on a substrate to determine the forces of the cell on the underlying pillars (Xiao et al, 2018). The well-defined shape of the pillars allows for easy computation of the forces applied by the cells. However, this technique cannot be used for 3D cell force measurements as the pillars are only 2.5D, and the deflections are in 2D. The forces on each pillar can be calculated by mapping the deformation of an array of pillars. This requires the pillars to have a known aspect ratio and Young’s modulus. Again, analytical and numerical methods can be employed to predict and verify the results.

Traction force microscopy (see Figure 4G) on the other hand, is based on the use of fluorescent beads inside an elastic substrate to assess the forces that cells apply to the ECM (Narasimhan et al, 2020). The cells can either grow on the substrate (2D force measurement) or grow in the substrate (3D force measurement). The position of the fluorescent beads compared to reference points can then be used to calculate the displacement and, thus, the forces of the cells. Optical fluorescence imaging techniques can be employed for the analysis of the samples.

## Applications of engineered microenvironments in neuromechanobiology

**FIGURE 5:**
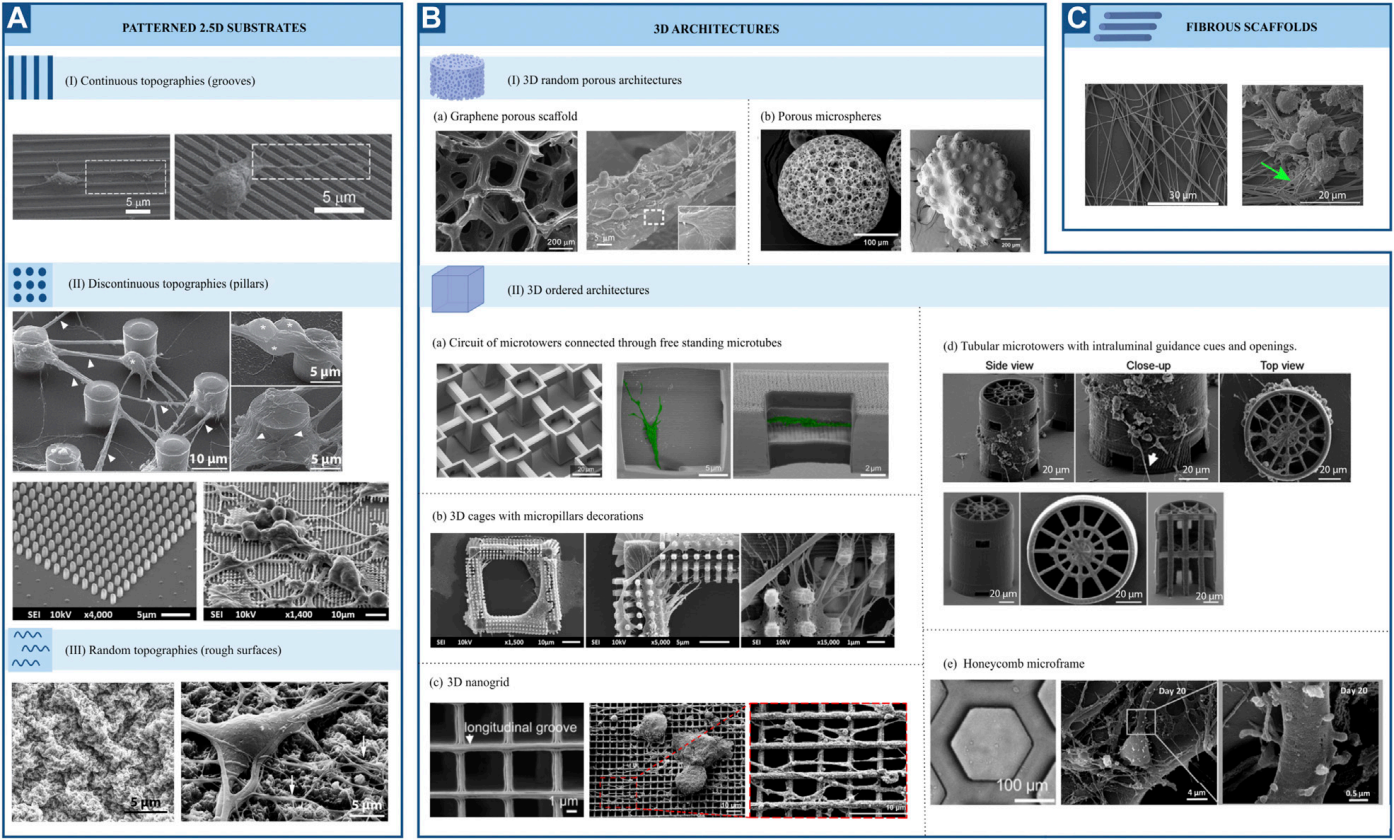
Representative SEM pictures of different topographies employed for neuromechanobiological studies (adapted end reproduced with permission from the mentioned references). **(A)** Patterned 2.5 substrates. **(I)** Continuous topographies (Fozdar et al, 2010), **(II)** Discontinuous topographies (Limongi et al, 2013; Sharaf et al, 2022) and **(III)** Random topographies (Cesca et al, 2014). **(B)** 3D architectures. **(I)** 3D random porous architectures **(a)** Li et al, 2013; **(b)** Sandhurst et al, 2022 **(II)** 3D ordered architectures **(a)** Harberts et al, 2020; **(b)** Sharaf et al, 2022; **(c)** Agrawal et al, 2021; **(d)** Turunen et al, 2017; **(e)** Left (phase contrast image): Huang et al, 2021 and middle and right: Koroleva et al, 2021. **(C)**. Fibrous scaffolds (Czeisler et al, 2016).

**FIGURE 6:**
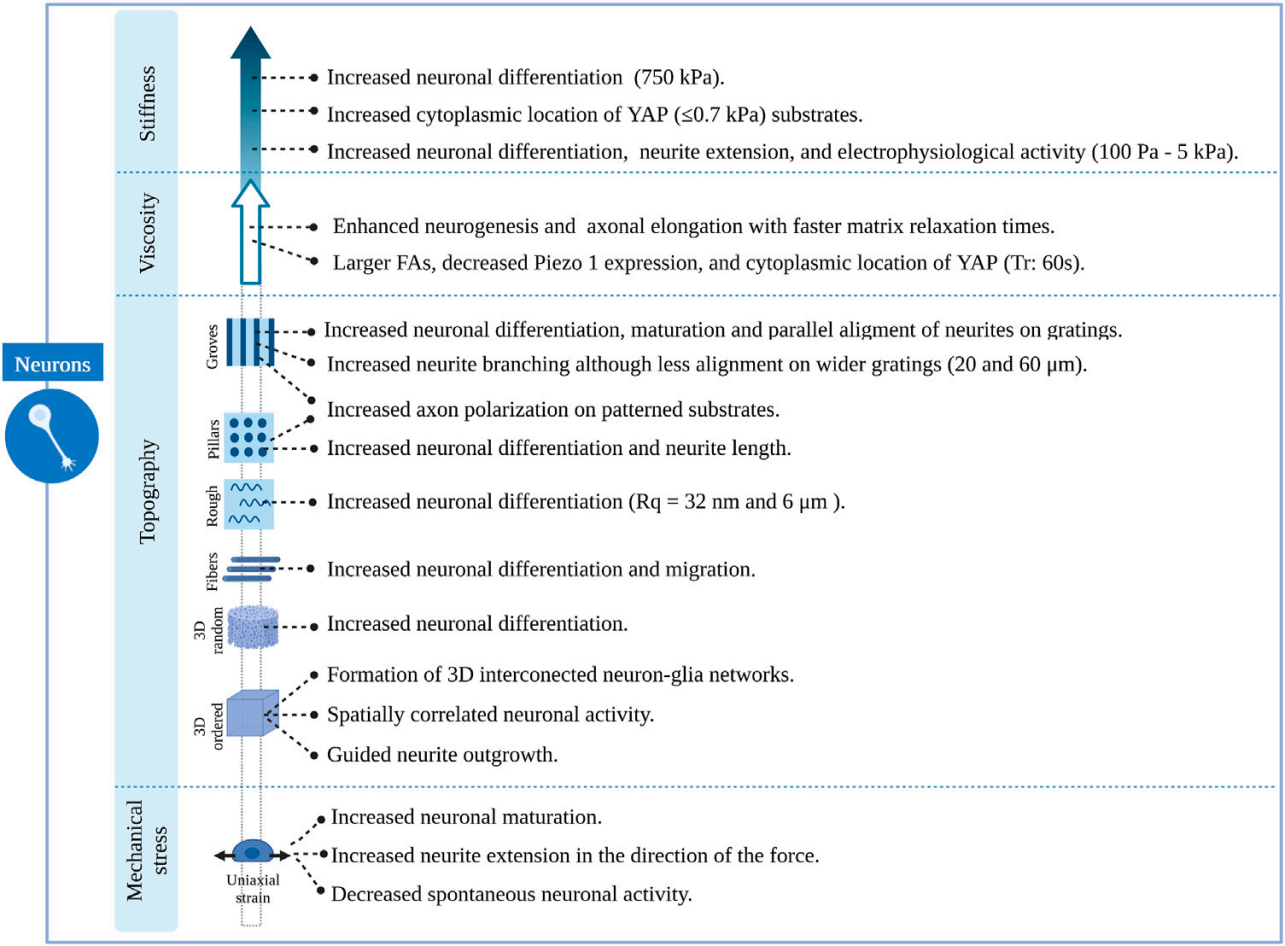
Overview of the effects induced by different features of engineered microenvironments on neuronal cells.

**FIGURE 7:**
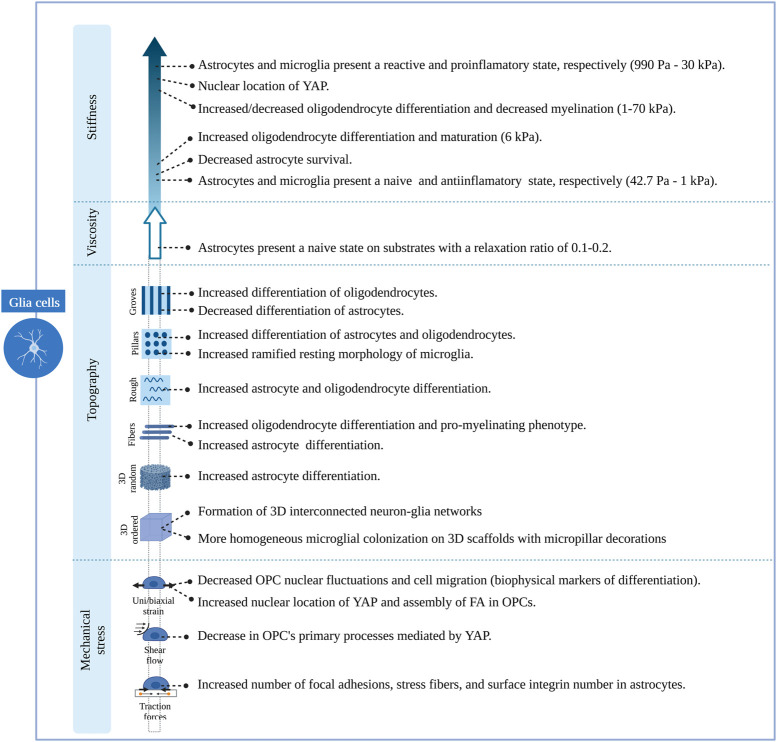
Overview of the effects induced by different features of engineered microenvironments on glial cells.

Understanding the cellular mechanisms of neuronal and glial cells and their role in the physiology and pathophysiology of the brain has represented a major challenge due to the lack of appropriate models/platforms to study mechanobiological processes. Nevertheless, remarkable progress has been achieved in the development of *in vitro* engineering cell culture systems, allowing a better understanding of neuromechanobiology. These platforms offer the possibility to tune the stiffness, viscoelasticity and topography of the culture environment, and to apply external forces to cells and examine their contribution to cell behaviours such as motility, proliferation, adhesion and proliferation (Marinval and Chew, 2021). In this section, we will discuss several research studies that illustrate the influence of stiffness, viscoelasticity and topography of *in vitro* engineered culture systems along with mechanical stresses on neuronal and glial cell phenotypes. These studies are summarized in Supplementary Tables S1, S2 and Figures 5–7.

### Topography of engineered microenvironments

The response of neural cells to surface topography has been extensively studied. Numerous *in vitro* studies have shown that topographical cues strongly influence cell fate, morphology, migration and function (Leclech and Villard, 2020). Patterned surface substrates can be classified into three large groups: continuous, discontinuous, and random topographies (Figure 5A). Grooves, ridges, gratings, and fibres can be classified as continuous features, while pillars, cones, holes, and posts constitute to discontinuous patterns. Lastly, micro and nano roughness comprise random topographies. Continuous and discontinuous patterns are further classified based on their directionality in anisotropic and isotropic features. The former provides cues along a single axis. In contrast, isotropic patterned substrates provide cues along multiple axes. For this reason, they can also be named unidirectional or multidirectional features, respectively (Leclech and Villard, 2020). Along with patterned substrates, fibrous scaffolds and 3D structures are also used (Figures 5B, C). There is already an extensive body of literature reviewing the effect of surface topography on neural cell differentiation and phenotype. Therefore, in this review, we will focus on the most relevant findings regarding this topic. For greater detail, the reader is referred to previous reviews (Simitzi et al, 2018; Eftekhari et al, 2020; Leclech and Villard, 2020; Sirkkunan et al, 2021). The results described in this section are summarized in Supplementary Table S1.

#### Continuous topographies

Several studies agree on the role of continuous anisotropic cues promoting neuronal over astroglial cell fate, either in the absence or presence of differentiation media. This effect was observed for both nano and micro-gratings, although it was more remarkable on nano-topographies (Ankam et al, 2013) and seems to be regulated by integrin-mediated activation of focal adhesion kinase. Human mesenchymal cells upregulated neuronal marker expression when cultured on nanogratings (250 nm), an effect that was abolished when using Rho or actomyosin contractility inhibitors. Remarkably, focal adhesions were significantly smaller and more elongated on grooves in the nanometric range than those on unpatterned substrates (Teo et al, 2013). In addition to an increase in neuronal cell fate, an increase in oligodendrocyte differentiation was observed on linear and circular micro-grooves (Qi et al, 2013). Nevertheless, the physical dimensions of grooves and ridges (depth, spacing and width) proved to be important for the morphology, branching and alignment of neural cells. Primary human neural progenitors cultured on deep (25 μm) gratings aligned along the topography but showed a different neurite response in terms of alignment and branching depending on the specific groove width. Narrow (5–10 μm) grooves constrain cells leading to unfavourable conditions for neuronal differentiation and fewer neurites, which are highly aligned. In contrast, culturing cells on wider (20–60 μm) grooves of the same depth resulted in higher neurite branching but less alignment, being comparable to that of cells cultured on flat surfaces (Béduer et al, 2012). Alternatively, narrower (300 nm and 2 μm) and shallow (450 nm) grooves (Figure 5AI) also favoured a more perpendicular alignment of neurons (Fozdar et al, 2010). This last type of guidance offers neurites multiple points of contact, similar to the guidance offered by continuous multidirectional and discontinuous topographies (Leclech and Villard, 2020). These studies indicate that neuronal phenotypes are favoured by topographies that present a clear directionality, such as grooves or ridges (Figure 6).

Biomimetic nanofibrous scaffolds, resembling the architecture of the natural extracellular matrix, have also been employed for many *in vitro* studies. Mouse neural stem cells cultured on aligned and randomly oriented electrospun nanofibers (Ø = 735 nm) showed an increase in cell viability and proliferation compared to unpatterned surfaces of the same material and glass coverslips (Hajiali et al, 2018). Moreover, these cells migrated following the fibres at a speed that was maximized in the direction of these cues, leading to a faster organization of 3D cellular networks. Additionally, they found an increased expression of neuronal markers and enhanced activity on neural progenitors cultured on fibrous scaffolds, indicating a greater neuronal differentiation (Hajiali et al, 2018). Interestingly, other studies showed that neural stem cells respond differently to random or aligned fibre organization. Culturing rodent adult neural stem cells on aligned nanofibers significantly enhances neuronal fate specification over randomly oriented fibres. Here, the relevance of fibre size was also emphasized, with the highest portion of neuronal differentiation found at 480 nm (Lim et al, 2010). Fiber-diameter dependent effects might be derived from different interaction mechanisms between cells and scaffold. Rodent neurons cultured on aligned ultrathin nanofibers (60 nm) expressed β1-integrin focal adhesions throughout the entire cell surface (cell soma and neurites), while those growing on aligned submicron-fibres (300 nm) formed these structures only on the cell soma (Mori et al, 2021). Czeisler et al showed how fibres (Figure 5C) with dimensions similar to radial glia (Ø = 1 μm) and small blood vessels (Ø = 10 μm), mimicking anatomical features that neural cells encounter during development, resulted in differential responses from rodent neural stem cells. Neurospheres plated on large fibers coated with Poly-d-lysine barely interacted with fibres and preserved a spherical morphology, while those plated onto small fibres with the same coating extended cellular processes showing a migratory morphology. Interestingly, they also observed that Rho inhibition reverses the anti-migratory effect of large fibres (Czeisler et al, 2016). Glial phenotypes seem to be affected by fibrous topographies as well. Rodent neural progenitor cells cultured on a commercial fibrous 3D scaffold composed of randomly distributed microfibers (Ø = 1,7 μm; mean inter distance = 10 μm) showed increased differentiation towards neurons, astrocytes and oligodendrocytes and increased promyelinating phenotype of the latter, compared to cells cultured on glass (Flagelli et al, 2021). Based on these results, it seems that fibres, similarly to grooves or ridges, offer directionality to neural stem cells, favouring neuronal differentiation. Interestingly, fibres also seem to promote glial differentiation in contrast to grooves o ridges (Figure 7).

#### Discontinuous topographies

Discontinuous topographies, such as pillars and wells with a size in the nano and micrometre range, have been proven to promote astrocyte differentiation while reducing neuronal and oligodendroglial fates (Ankam et al, 2013). Here again, the physical dimensions of discontinuous features, such as shape and distribution, are key to understanding the effect of these structures on cell phenotype. The results of this study demonstrate that discontinuous topographies mainly promote astroglia differentiation. However, their precise dimensions, number, and distribution are relevant to this effect, and depending on them, discontinuous topographies might promote neuronal differentiation as well (Figures 6, 7). For example, Park et al (Park et al, 2016) demonstrated that homogenously distributed micropillar arrays promoted neurite extension in several directions, while if micropillars are spaced so that they line up in parallel rows, a rather unidirectional guidance of neurite outgrowth was observed. Another relevant aspect to consider is the spacing between these features: sparse arrays of pillars allow neurites to grow between them, while on dense arrays, cells and their neurites will navigate on top of the structure (Leclech and Villard, 2020). In fact, rodent neural stem cells cultured on dense nanopillar arrays grew and extended their neurites over these structures. Notably, neurite outgrowth did not show any directionality, but neuronal differentiation was promoted on nanopillar arrays over control flat surfaces (Lee et al, 2020). Limongi et al (Limongi et al, 2013) showed that neurons grown on pillars with nanopattered sidewalls displayed a higher survival rate compared to standard cultures and that they developed mature networks with physiological excitability (Figure 5AII, top). Lastly, the microglia phenotype seems also to be modulated by discontinuous topographies. Sharaf et al demonstrated that primary primate microglia displayed an increased ramified resting phenotype when cultured on micropillars arrays (Figure 5AII, bottom) when compared to stiff, flat surfaces (Sharaf et al, 2022).

#### Random topographies

Little research has been conducted to study the effect of random topographies on neural fate and behaviour. Random topographies are stochastic surfaces defined by roughness parameters (R_q_) (Simitzi et al, 2018). Differentiation of human iPSCs towards a neuronal fate was promoted on intermediate micro-roughness surfaces (R_q_ = 6 μm), compared to flat (R_q_ = 0.3 μm) or more rough (R_q_ = 38 μm) surfaces (Li et al, 2016). Human stem cells are also sensitive to nanoscale roughness surfaces: smooth surfaces (R_q_ = 1 nm) favoured adhesion and self-renewal of human ESCs, while rough surfaces (R_q_ = 70 and 150 nm) promoted their spontaneous differentiation. These events were linked to a differential distribution of focal adhesions, which were observed on the periphery of cells on smooth surfaces and throughout the complete cell spreading area on patterns creating a nano-roughness (Chen et al, 2012). The involvement of focal adhesions in nano-roughness surfaces-mediated neural differentiation was also demonstrated by Chen et al They observed that ultra-nanocrystalline diamond sheets induced the spontaneous differentiation of rodent neural stem cells into neurons, astrocytes and oligodendrocytes, an effect that was abolished after blocking integrin β1 (Chen et al, 2010). Cesca et al (Cesca et al, 2014) showed that randomly nanopatterned 3D poly-ε-caprolactone (PCL) film with poly-d-lysine coating improves the differentiation of neurons compared to flat PCL films (Figure 5AIII).

Lastly, stretch-activated cation channels like Piezo-1 have also proved to be relevant for nano-roughness mechanosensing, as shown by Blumenthal et al (Blumenthal et al, 2014). They mimicked random ECM nanoroughness using an assembly of monodispersed silica colloids of increasing size (R_q_ from 12 to 80 nm). Neuronal morphology and function of PC12 cells, as well as neuronal linage commitment of rodent neural stem cells, were favoured at a surface roughness of R_q_ = 32 μm; while the interaction between primary rodent neurons and astrocytes was promoted on roughness higher and lower than 32 μm. Notably, all these effects were abolished after the inhibition of Piezo-1 (Blumenthal et al, 2014) (Figures 6, 7). Simitzi et al (Simitzi et al, 2015) found that low and intermediate rough microcone surfaces supported PC12 cell differentiation, whereas highly rough ones (large distances between microcones) did not. This highlights the relevance of surface roughness optimization.

In conclusion, random topographies can promote both neuronal and glial fates. Their specific effect could depend on the degree of roughness of the surface, but further studies are needed to evaluate this.

#### 3D architectures

More complex 3D architectures, mainly porous scaffolds, have been employed to study the interaction of neural cells with their physical environment. 3D graphene porous scaffolds (Figure 5BIa) promoted the proliferation and differentiation of rodent neural stem cells towards neurons and astrocytes, while no significant difference in oligodendrocyte differentiation was observed when compared to cells cultured on 2D graphene films (Li et al, 2013). Similarly, Guo et al (Guo et al, 2021) cultured rodent neural stem cells on 3D graphene porous scaffolds embedded in a cellulose polymer. Notably, they observed an enhanced proliferation when these cells were cultured in proliferation media on graphene-cellulose scaffolds, compared to graphene scaffolds only. They also observed an increase in neuronal differentiation when cultured in differentiation conditions on graphene-cellulose scaffolds. Interestingly this was accompanied by a reduced expression of focal adhesion proteins mRNA on these scaffolds, compared with analogous structures without cellulose (Guo et al, 2021). Likewise, microspheres scaffolds (Figure 5BIb) have been employed to culture human iPSC-derived neural progenitor cells and were found to promote both neuronal and glial differentiation (Sandhurst et al, 2022). Silk fibroid-based biomaterials also present an opportunity for *in vitro* 3D neural cultures. 3D neuronal networks were achieved using a doughnut-shaped porous silk sponge, where neuronal projections grew within a collagen-filled central region (Chwalek et al, 2015). Therefore, random 3D structures seem to promote neuronal and glial differentiation equally (Figures 6, 7).

In contrast to these random, porous microstructures, engineers have designed ordered structures that are highly reproducible. Using direct laser writing by two-photon polymerization, Fendler et al (Fendler et al, 2019) fabricated a circuit of micro towers connected through free-standing microtubes, resembling the configuration of myelin sheets with areas of high and low confinement alternatively. They were able to guide neurite outgrowth of cultured primary rodent neuronal cells along established paths on the scaffolds, building tailor-made neuronal networks. Furthermore, they showed that these cells display electrophysiological activities similar to those cultured on control substrates (Fendler et al, 2019). Similarly, Harberts et al used a comparable scaffold (Figure 5BIIa) to culture human iPSC-derived neurons, observing guided neurite outgrowth and neuronal electrophysiological activity as well (Harberts et al, 2020, 2021). Huang et al (Huang et al, 2021) also created neural networks with the aid of ordered scaffolds. They seeded human iPSC-derived neural progenitors and human endothelial cells in a honeycomb micro-frame (Figure 5BIIe, left) covered with gelatin microfibers and observed the formation of interconnected 3D neural clusters with high expression of neuronal, astrocytic and synaptic markers that displayed spatially correlated neuronal activity (Huang et al, 2021). Honeycomb-based scaffolds (Figure 5BIIe, middle and right) were also used by Koroleva et al (Koroleva et al, 2021), who created complex neuron-glia networks containing neurons of all six cortical layers, different types of interneurons and astrocytes derived from human iPSCs. These networks were viable for long periods of time, in contrast to their 2D counterparts, and presented spontaneous neuronal activity. Likewise, Turunen et al (Turunen et al, 2017) built tubular microtowers that supported neuronal network formation and neurite orientation of human iPSC-derived neurons (Figure 5BIId). Guided neurite outgrowth was also observed on primary rodent neurons cultured on 3D nanogrids (Agrawal et al, 2021). Finally, 3D structures have also been employed to study the phenotype of primary primate microglial cells. Sharaf et al (Sharaf et al, 2022) observed that microglia presented various morphologies when cultured on 3D cages (Figure 5BIIb) and the colonization of the cages was more homogeneous in the presence of nano and micropillars decoration. In summary, the easy tuning of their geometrical features has proved the versatility of designed ordered 3D scaffolds. These structures have shown to be able to steer *in vitro* neuronal circuits and promote the maturation of neuron-astrocyte networks (Figures 6, 7). Nevertheless, further work is needed to understand their effect on other glial cell types.

Ultimately, the combination of physical dimensions of each scaffold might initiate specific downstream signalling pathways and regulate cell phenotype in different ways. Consequently, comparisons of different studies need to consider the specific physical dimensions of apparently similar topographies since these can be key for explaining the differences in cell behaviour. It is also worth mentioning that different topographies may have distinct effects on the initial lineage commitment and subsequent maturation stages of neural cells. Micro-grating surfaces showed to be favourable for the initial neuronal lineage commitment of human iPSCs, while micropillar arrays promoted a greater branching complexity and neuronal activity, being beneficial for later maturation stages (Tan et al, 2018). In line with the consecutive application of topographies, the mechanical memory of cells must also be considered. This property allows cells to store information about their past mechanical environment (Kanoldt et al, 2019). The mechanical memory of neural cells is illustrated by the preservation of the neuronal morphology and functional phenotype of human neural stem cells cultured on patterned substrates and transferred to a flat surface (Yang et al, 2014). Therefore, when examining the interaction between cells and the scaffold’s topographical features, it is essential to consider their specific combination of shapes, dimensions and distribution and the particular stage of differentiation or maturation that neural cells are at.

### Mechanical properties of engineered microenvironments

#### Stiffness

As mentioned before, the stiffness of cells and their environment are major regulators of cell behaviour (Marinval and Chew, 2021). In fact, substrate stiffness acts as an instructive cue for lineage commitment. Keung et al (Keung et al, 2012) cultured human ESCs and iPSCs in polyacrylamide gels of different stiffness and found that a higher proportion of cells differentiated towards a neuroectodermal and neuronal lineage when cultured on substrates with stiffness values similar to those of the brain (100 and 700 Pa). These results are in line with other work performed on human stem cells. Human ESCs were found to differentiate into neurons in polyacrylamide gels of 700 Pa in the presence of soluble factors that promote a pluripotent cell state. Neuronal differentiation was, therefore, mediated exclusively by mechanical cues, and it was linked to a decrease in the polymerization of F-actin and translocation of YAP to the cytoplasm (Musah et al, 2014). Similarly, human ESCs cultured on PDMS substrates showed increased neuronal differentiation on compliant surfaces. Again, YAP cytoplasmic location and depolymerization of the cell actin cytoskeleton were linked to neuronal differentiation. Furthermore, this study showed that the Hippo-pathway mediated YAP location. Additionally, they found evidence of mechanoregulation of neural subtypes since the expression of anterior and posterior patterning genes was responsive to varying stiffness (Sun et al, 2014). Studies on rodent cells support these findings and link other mechanotransducer molecules to stiffness-mediated cell fate decisions, such as β1 integrin (Du et al, 2011), the RhoA protein and CDC42 (Keung et al, 2011) (Figure 6). These studies also identified substrate stiffness as a modulator of astrocytic differentiation, with stiffer substrates promoting astrocytic differentiation and survival (Saha et al, 2008; Keung et al, 2012). The stretch-activated ion channel Piezo 1 has also been linked to cell fate decisions. On the other hand, primary human neural progenitor cells (NPCs) cultured on stiff silicone gels were found to promote differentiation into neurons more than softer substrates. Cells cultured on stiff substrates displayed a higher Piezo 1 activity and nuclear location of YAP. Interestingly, pharmacological or genetic inhibition of Piezo 1 resulted in increased astrocytic differentiation (Pathak et al, 2014). These results oppose the observations of the abovementioned studies, where neuronal differentiation was promoted on compliant substrates. Differentiation of neural stem cells into oligodendrocytes also seems to be related to substrate stiffness. Rodent primary oligodendrocyte precursor cells (OPCs) culture on polyacrylamide gels differentiated into mature oligodendrocytes on stiffer substrates. Notably, OPCs stiffen during differentiation, regardless of substrate stiffness (Jagielska et al, 2012). These cells showed the same response when cultured on fibrous substrates of different stiffness, differentiating into mature oligodendrocytes with increased intrinsic fibre stiffness. It is worth mentioning that YAP was not involved in oligodendrocyte differentiation (Ong et al, 2020). Contrary to these results, other studies found soft substrates to promote oligodendrocyte differentiation. They also highlighted the potential role of YAP in oligodendrocyte differentiation, as this protein is mainly located in the nucleus when cells are cultured on stiff substrates (Lourenço et al, 2016; Urbanski et al, 2016). Lastly, Sharaf et al (Sharaf et al, 2022) observed that microglia morphology was affected by the effective shear module of pillar-arrays, being the morphology of these cells more ramified when cultured on low effective shear stress modulus surfaces (Figure 7).

Mechanical cues are known not only to modulate cell differentiation but also cell function and morphology. Human iPSC (hiPSC)-derived neural stem cells cultured on polyethylene glycol (PEG) hydrogels extend longer neurites in softer substrates, but no discrepancies were found in the expression of neuronal differentiation markers across stiffness (Mosley et al, 2017). Remarkably, specific subtypes of neurons respond differently to substrate stiffness. While hiPSC-derived forebrain neurons prefer softer substrates to extend their neurites, hiPSC-derived motor neurons do it preferentially in stiffer substrates. For the latter, this behaviour was linked to increased activity of RhoA, myosin and FAK proteins (Nichol IV et al, 2019). Neuronal activity is also regulated by this mechanical cue. Human ESC displayed electrophysiological activities comparable to those of primary neurons *in vivo* when cultured on soft PDMS substrates (Sun et al, 2014). Similarly, human ESCs cultured on soft polyacrylamide gels showed spontaneous postsynaptic currents and action potentials earlier than those cultured by standard protocols (Musah et al, 2014) (Figure 6).

Substrate stiffness can also influence the morphology and reactive state of astrocytes. Human astrocytes exhibit an extended morphology with increased substrate stiffness, along with increased traction, strain energy and intracellular stress (Bizanti et al, 2021). These results are in accordance with observations on rodent astrocytes. The latter also displayed an extended and hypertrophic morphology on stiff substrates, where they also upregulated the expression of inflammatory genes and proteins. Higher expression and nuclear localization of YAP were found in astrocytes cultured on stiff substrates. Thus, YAP was hypothesized as the mediator between substrate stiffness and astrocytic activation (Moshayedi et al, 2014; Hu et al, 2021). Moshayedi et al also observed a comparable performance on rat primary microglia cultured on polyacrylamide gels: these cells upregulated their inflammatory phenotype on stiff substrates. In line with these findings, rat primary microglia presented a round morphology and upregulated the expression of anti-inflammatory makers when culture on compliant substrates *via* the activation of stretch-dependent chloride channels (Blaschke et al, 2020). Remarkably, rat primary microglia migrate preferentially towards stiff substrates and exert forces on the substrate that increase with substrate stiffness (Bollmann et al, 2015). Finally, hiPSC-derived oligodendrocytes displayed increased migration with increased stiffness (Espinosa-Hoyos et al, 2020). The myelinating capacity of rat primary OPC was also found to be modulated by substrate stiffness, decreasing on stiff substrates (Ong et al, 2020) (Figure 7). The results described in this section are summarized in Supplementary Table S2.

The variations between the reported effects of substrate compliance on cell fate, morphology and function among different studies might be due to differences in cell culture materials (substrate composition, tested range of stiffness, cell culture media) and the cells employed (human or rodent origin, maturation stage). A better understanding of the molecular players and pathways involved in stiffness mechanotransduction on different brain cell types might help to elucidate these disagreements.

#### Viscosity

The effect of substrate viscosity on the behaviour of brain cells is largely unexplored, especially compared to substrate stiffness and topography. Chen et al (Chen et al, 2021) cultured rat neuronal lineage cells on hyaluronan hydrogels with different relaxation times. Enhanced neurogenesis and increased axonal length were observed when neuronal cells were cultured on substrates with shorter relaxation times. These changes were related to larger FAs, decreased Piezo-1 expression and cytoplasmic location of YAP (Chen et al, 2021) (Figure 6). Similarly, the expression of GFAP of primary human fetal-derived astrocytes cultured on collagen/hyaluronic acid hydrogels was strongly correlated with the gel’s relaxation rate (Placone et al, 2015) (Figure 7). The remarkable lack of literature on this topic points out the opportunity for research in this field that will bring us closer to understanding brain cells’ interaction with their biophysical microenvironment. A summary of these studies can be found in Supplementary Table S2.

#### Mechanical stress

Applied mechanical forces have been demonstrated to influence neuronal and glial cell behaviour. Makhija et al cultured rat OPCs on PDMS elastomeric plates and applied uniaxial strain to these cells. They demonstrated that mechanical strain decreased biophysical markers of oligodendrocyte differentiation, such as nuclear fluctuations and cell migration, along with increased expression of tubulin, which is also related to OPC differentiation (Makhija et al, 2018). The uniaxial strain was also proved to induce nuclear localization of YAP as well as assembly of focal adhesions on rat OPC cultured on silicone elastomeric plates. YAP was not related, in this case, to oligodendrocyte differentiation. The same authors tested the effect of shear stress on rat OPC and observed a decrease in the oligodendrocyte primary process mediated by YAP (Shimizu et al, 2017). Similarly, Arulmoli et al (Arulmoli et al, 2015) applied static stress to rodent neural progenitor cells plated on rubber membranes coated with laminin and observed a reduced oligodendrocytic differentiation mediated by integrin binding to the culture surface. Rat astrocytes are also susceptible to mechanical stresses. Pérez et al (Pérez et al, 2021) used a pull-up traction assay based on magnetic beads coated with integrin-ligand proteins. The application of traction forces on astrocytes led to an increase in integrin surface expression, focal adhesions and stress fibres. They also found that the rearrangement of the cytoskeleton of astrocytes was able to generate traction forces on the substrate from within the cell, using integrins as mediators. Likewise, Li et al (Li et al, 2022) applied biaxial stretch to primary rodent astrocytes and observed changes in protein expression and signalling pathways that might be associated with astrocyte activation (Figure 7). Furthermore, the uniaxial strain was found to modulate the mechanical properties and behaviour of hiPSC-derived motor neurons. These cells were subject to strain forces on a PDMS stretching device, inducing the fluidization of cell membranes as well as a decrease in spontaneous neuronal activity (Bianchi et al, 2019). Conversely, Chang et al (Chang et al, 2013) observed that uniaxial strain increases neuronal differentiation and maturation of rat neural stem cells, along with the alignment of their neurites on a PDMS stretching device. Similarly, PC12 cells reoriented themselves to the perpendicular direction of the applied stretch forces (Lin et al, 2020). Interestingly, Minegishi et al found that traction forces on the growth cone of primary rodent neurons induce the advance of this leading process, which was mediated by the axonal clutch molecule Shootin1b (Minegishi et al, 2018).

Lastly, the uniaxial strain was found to stimulate rapid amyloidogenic processing of the amyloid precursor protein on human iPSC-derived neurons form cognitively unimpaired individuals (Chaves et al, 2021) (Figure 6). The results of these studies highlight mechanical forces imposed on neural cells as significant modulators of their behaviour. Notably, many of these studies focus on oligodendroglia cells, while the effect of mechanical stresses on neurons, astrocytes and microglia have been studied to a lesser extent. Importantly, the majority of these conclusions have been drawn from studies on rodent cells, which emphasizes the relevance of analysing these events on human cells. Refer to Supplementary Table S1 for a summary of these studies.

## Discussion

A growing body of evidence draws attention to the strong influence of mechanical and topographical cues on neural cell behaviour, and its ability to modulate cell morphology, differentiation and phenotype. Progress in biomaterial engineering and microfabrication methods has created the opportunity to design and customize architectures to recapitulate some of the *in vivo* brain’s features, providing cells with a more physiologically relevant microenvironment. This enables to study the two-way interaction between cells and their physical surroundings.

Interestingly, most of our current understanding of how neural cells sense and respond to mechanical and topographical cues comes from cells in 2D culture modes. Nevertheless, it is widely recognized that neural cell behaviour in 2D culture formats presents an altered morphology and gene expression, which differs significantly from that displayed in more physiological 2.5D and 3D environments. Furthermore, 2.5D and 3D-engineered culture systems were recently employed to identify important mechano-related signalling pathways and to study differential cell behaviours elicited by diverse physical cues. Consequently, although some mechanobiological processes may carry over from 2D to 2.5D or 3D environments, validating the findings in a 3D setting is necessary.

In light of this, recent efforts in the field have focussed on addressing biomechanical studies with 3D culture platforms that more closely resemble the mechanical and topographical conditions of the *in vivo* neural environment. Unordered-porous scaffolds, such as hydrogels, have been widely employed for this purpose. Although they provide a very relevant and physiological culture platform, these scaffolds are hindered by reduced reproducibility due to variability in their structure. The advent of microfabrication techniques, such as 3D printing, allows to manufacture ordered and reproducible scaffolds, which may help to overcome these limitations. 3D structures with an ordered geometry can provide the biological relevance of 3D cultures together with the high reproducibility characteristic of 2D settings. Hence, a major challenge for future works is the design of multiscale 3D structures with tuneable stiffness that incorporate and combine the micro and nano-topographical features already studied on 2.5D platforms to evoke the desired response on neural cells. The (nano) mechanical properties of the (micro) environment, in which neural cells grow, deterministically influence their phenotype and can help us model different aspects of the brain in health and disease. Therefore, it is important to consider how the different properties of the scaffold would influence cell function and understand what combination of mechanical cues would be sufficient to recapitulate key aspects of the brain. Altogether, this will enable us to design scaffolds according to a targeted application (disease modelling, tissue regeneration, etc.). Similarly, the study of cell mechanical properties and the forces exerted by cells on their surroundings are usually performed on single cells on 2D substrates, which might not be a faithful representation of how forces are transmitted within the *in vivo* 3D microenvironment. Again, future research is required to bridge our current knowledge from 2D studies with neural cells cultured on more complex 3D-engineered scaffolds.

Furthermore, a large number of neuromechanobiological studies have been performed with animal cells, while only a few have used human stem cells. Notably, combining human stem cells and biomaterial engineering methods will open unique opportunities to build relevant human brain models. In any case, the variability of stem cell types, origin and differentiation methods used in previous neuromechanobiology studies hinders the comparability of their findings, leading to contradictory results on some occasions. Therefore, having more comparable conditions would help to advance our understanding of the biomechanical interactions of neural cells and their environment. Of particular interest are iPSCs, which, in contrast to ESCs, are generated from adult human somatic cells, thus having very little or no ethical drawbacks. In addition, iPSCs allow the derivation of any specific relevant cell type from both healthy and diseased individuals, maintaining the genetic information. Therefore, iPSCs are promising to be a powerful tool for studying mechano-related disease mechanisms and identifying potential therapeutic targets. Interestingly, measurements of the forces of the cells on microfabricated structures could be performed to determine the difference in mechanobiology of both healthy and diseased cells.

Lastly, an appealing research path to be explored in the near future is the combination of scaffold-free models such as iPSC-derived brain organoids and scaffold-based strategies. The self-organizing properties of organoids, which create brain tissue-like structures, can be merged with designed microenvironments providing biomechanical constraints. Importantly, 3D-engineered scaffolds can help to address two of the main challenges in human iPSC-derived brain organoids technology: the cell population heterogeneity derived from differentiation protocols that mainly rely on biochemical cues, and the formation of early necrotic cores, which are detrimental for long-term studies. Reproducible 3D engineered structures could help, therefore, by guiding linage specification.

In conclusion, engineered neural microenvironments, in combination with iPSC technology, can be used as tools to build more complex brain models of higher physiological relevance, helping to identify new targets for therapeutic intervention and serving as drug-testing platforms to validate these findings. We envision that two pathways for iPSC-derived neural cell studies can be explored in the future. First, we envision that engineered scaffolds can mimic the 3D microenvironment while promoting faster differentiation to create improved human and disease model systems. Second, the manufactured structures can be used to measure the biomechanical properties of healthy *versus* diseased neural cell types, potentially providing new approaches to identify disease-specific markers that are predictive for pathological states. Thus, recapitulation of key aspects of *in vivo* neural microenvironment in 3D culture models is essential to provide physiologically relevant results, which can be employed for diagnostic purposes and finding new therapeutic targets.
