# Supplementary material for: Engineered cell culture microenvironments for mechanobiology studies of brain neural cells
Source: Front Bioeng Biotechnol. 2022 Dec 14;10:1096054. doi: 10.3389/fbioe.2022.1096054 (PMC9794772; doi:10.3389/fbioe.2022.1096054)
Supplement: Supplementary file 1 [file DataSheet1.PDF]

Supplementary Material

Engineered cell culture microenvironments for mechanobiology studies of brain neural cells

Lucía Castillo Ransanz[1]†, Pieter F.J. van Altena[2]†, Vivi M. Heine[1,3]*, Angelo Accardo[2]*

[1]Department of Child and Adolescence Psychiatry, Amsterdam Neuroscience, Emma Children's Hospital, Amsterdam UMC Location Vrije Universiteit Amsterdam, Amsterdam, The Netherlands.

[2] Department of Precision and Microsystems Engineering, Delft University of Technology, Delft, Netherlands

[3]Center for Neurogenomics and Cognitive Research, Vrije Universiteit Amsterdam, Amsterdam Neuroscience, Department of Complex Trait Genetics, Amsterdam UMC Location Vrije Universiteit Amsterdam, Amsterdam, The Netherlands.

† These authors contributed equally to this work and share first authorship

* These authors contributed equally to this work and share corresponding authorship

Correspondence:
Corresponding Authors
A.Accardo@tudelft.nl
vm.heine@amsterdamumc.nl

Supplementary Table 1. Applications of engineered microenvironments in neuromechanobiology: topography and mechanical stresses

General mechanical property or stimulation	Mechanical property/ stimulation type	Substrate features	Material and fabrication technique	Cell type	Culture media	Mechanistic study	Biological outcome description	Ref.
Topography	Patterned 2.5D substrates (continuous and discontinuous topographies)	Continuous:<br>• Nano-gratings (width:250nm).<br>• Micro-gratings (width:2µm).<br><br>Discontinuous:<br>• Micropillars (height:1µm).<br>• Microwells (depth:2µm).	PDMS (soft lithography)	Human ESC	Neuronal differentiation media	None	• **Neuron:astrocyte ratio** (Tuj1:GFAP) is **increased** in micro and nano-gratings compared to pillar patterns.<br>• **Higher neuronal maturation** (MAP2) and **longer aligned neurites** on nano-gratings compared to micro-gratings, pillars and unpatterned substrate.<br>• **Increased glial differentiation** on micropillars and wells (GFAP, Olig1).	Ankam et al., 2013
		Continuous:<br>• Nano-gratings (width:250nm).<br>• Micro-gratings (width:1,2 and 10µm).<br>• Unpatterned substrates	PDMS (soft lithography)	Human MSC	Neuronal differentiation media	p-FAK; Paxilin; F-actin	• hMSC showed an **increased expression of neuronal markers (MAP2, NF-H)** when cultured on nanogratings, compared to unpatterned substrates.<br>• Topography-induced **neuronal differentiation** of hMSC was dependent on **FAK phosphorylation.**	Teo et al., 2013
		Continuous:<br>• Circular micro-gratings (width: 2 and 10µm).<br>• Linear micro-gratings (width: 2 and 10µm).<br><br>Discontinuous:<br>• Micropillars (width: 2 and 10µm).	Silicon (photolithography)	Rodent adult NSC	Neuronal and glial differentiation media	MAPK/ERK	• **Increased neuronal differentiation** (Tuj-1) on circular and linear microgratings compared to flat controls.<br>• **Decreased astrocytic differentiation** (GFAP) on circular and linear microgratins compared to flat controls<br>• **Increased oligodendrocyte differentiation** (RIP) on circular and linear microgratings compared to flat controls.<br>• No significant differences in the differentiation towards neuronal or glial lineages between flat and micropatterned surfaces.	Qi et al., 2013
		Continuous:<br>• Micro-gratings (depth: 25µm).<br>○ Narrow: width= 5 and 10µm.<br>○ Wide: width= 20 and 60µm.	PDMS (soft lithography)	Primary human neural progenitors	Neuronal differentiation media	None	• Neural progenitors **showed increased neurite branching although less alignment** on wider gratings (20 and 60µm) compared to the narrower ones (5 and 10µm)	Béduer et al., 2012

Supplementary Table 1. Applications of engineered microenvironments in neuromechanobiology: topography and mechanical stresses (Continued)								
**Topography**	**Patterned 2.5D substrates (continuous and discontinuous topographies)**	Continuous:<br>• Nano-gratings (width:300nm).<br>• Micro-gratings (width:2µm).<br><br>Discontinuous:<br>• Nano-holes (width:300nm).<br>• Micro-holes (width:2µm).	PDMS (soft lithography)	Rodent embryonic neurons	Neuronal maintenance media	None	• **Axon polarization** of neuronal cells was significantly **higher** in <u>patterned substrates</u> than in the flat control, but no differences between topographies were found.<br>• **Neurons** presented **longer axons** on <u>2µm grooves.</u><br>• <u>Micro and nano gratings</u> mainly favoured a **parallel alignment** of neurites, although a **perpendicular orientation** was also observed.<br>• On the <u>300nm gratings</u>, neurites with perpendicular orientation were longer.	Fozdar et al., 2010
**Topography**	**Patterned 2.5D substrates (continuous and discontinuous topographies)**	Discontinuous:<br>• Micro-pillars (width:1µm; height: 1µm).<br>○ Interpillar distances: 3, 6, 9 and 12 µm.	Silicone (photolithography)	Rodent primary hippocampal neurons	Neuronal maintenance media	None	• <u>Homogenously distributed</u> micropillar arrays promoted **neurite extension in several directions,**<br>• <u>Spaced micropillars</u> lining up in <u>parallel</u> rows promoted a rather **unidirectional guidance of neurite outgrowth.**	Park et al., 2016
**Topography**	**Patterned 2.5D substrates (continuous and discontinuous topographies)**	Discontinuous:<br>• Nano-pillars (width:500nm; height: 1µm).	PDMS (soft lithography)	Mouse neural stem cells	Neuronal maintenance media	None	• **Increased neuronal differentiation** (Tuj-1) **and neurite length** when cultured on <u>nano-pillar arrays</u> compared to cells cultured on standard plates.	Lee et al., 2020
**Topography**	**Patterned 2.5D substrates (continuous and discontinuous topographies)**	Continuous:<br>• Micro-gratings (width:2µm).<br><br>Discontinuous:<br>• Micro-pillars (width:2µm; height: 2µm).	PDMS (soft lithography)	Human iPSC	Neuronal differentiation media	None	• <u>**Micrograted**</u> surfaces showed to be **favourable for the initial neuronal linage commitment** of hiPSCs**,** while <u>micropillar</u> arrays **promoted a greater branching complexity and neuronal activity**, being beneficial for **later maturation stages**	Tan et al., 2018
**Topography**	**Patterned 2.5D substrates (continuous and discontinuous topographies)**	Discontinuous:<br>• Micro-pillars (width:1µm; height:2.5 µm).	IP-Dip (2PP-DLW)	Primary primate microglia	Microglial culture media	None	• Micropillars promoted a ramified resting morphology of microglia cells compared to cells cultured on flat, stiff substrates.	Sharaf et al., 2022
**Topography**	**Fibrous scaffolds**	Electrospun nanofibers<br>• Ǿ = 735nm<br>• Random or aligned orientation	Polyphenylene sulfone (PPSu) (electrospinning)	Mouse neural stem cells	Neuronal differentiation media	None	• Neuronal cells showed an **increased viability, proliferation and migration speed** on <u>fibrous scaffolds</u> compared to PPSu films and glass coverslips.<br>• **Increased neuronal differentiation** (Tuj-1, DCX) **and activity** on <u>fibrous scaffolds</u> compared to PPSu films and glass coverslips.	Hajiali et al., 2018

Supplementary Table 1. Applications of engineered microenvironments in neuromechanobiology: topography and mechanical stresses (Continued)

**Topography**	**Fibrous scaffolds**	Electrospun nanofibers<br>• Ǿ = 260, 480 and 930nm<br>• Random or aligned orientation	Polycaprolactone (PCL) (electrospinning)	Rodent adult neural stem	Neuronal differentiation media	None	• **Neuronal differentiation** (Tuj-1) is **higher** in aligned than random fibres.<br>• **Highest proportion of neurons** (Tuj-1) was found on 480nm aligned fibres.	Lim et al., 2010
		Electrospun nanofibers<br>• Ǿ = 60 and 300nm<br>• Random or aligned orientation	Nylon (electrospinning)	Rodent neural progenitors	Neuronal differentiation media	β1-integrin; Vinculin; F-actin	• Neurons showed **greater neurite elongation** on fibrous scaffolds compared to nylon films.<br>• Neurite **branching** was **increased** on random nanofibers.<br>• **Vinculin-containing FA** established by neurons on both 60 and 300nm showed a **decreased number and size** compared to flat surfaces.<br>• **Integrin-containing FA** established by neurons on 60nm fibres were found throughout the **entire cell surface**, while those formed on 300nm fibres were localized at the **soma**.	Mori et al., 2021
		Electrospun nanofibers<br>• Ǿ = 1 and 10µm<br>• Random or aligned orientation	Polycaprolactone (PCL) (electrospinning)	Rodent neural progenitors	Neuronal differentiation media	ROCK	• Neurospheres plated on 10 µm fibres coated **barely interacted** with them and preserved a **spherical morphology**,<br>• Neurospheres plated on 1 µm fibres **extended cellular process** showing a **migratory morphology**.<br>• **Rho inhibition reverses the anti-migratory effect** of 10µm fibres	Czeisler et al., 2016
		Commercial fibrous scaffold: VITVO<br>• Ǿ = 1,7 µm<br>• mean interdistance = 10 µm<br>• Random orientation	Polybutylene terephthalate (PBT)	Rodent neural progenitors	Neural proliferation media	None	• Neural progenitors present an **increased differentiation towards neurons** (Tuj-1)**, astrocytes** (GFAP) **and oligodendrocytes** (MBP, CNPase) on fibrous scaffolds.<br>• Fibrous scaffolds favoured a **promyelinating phenotype of oligodendrocytes** (MBP, CNPase), compared to cells cultured on glass.	Flagelli et al., 2021
**Topography**	**Random topographies**	Micro-roughness surfaces<br>• R0 (flat): Rq= 0.3 µm<br>• R1: Rq= 6 µm<br>• R2: Rq= 38 µm	Polystyrene (injection moulding)	Human iPSC	Neuronal differentiation media	None	• **Higher percentage of neuronal differentiation** was observed on R1 surfaces.	Li et al., 2016

Supplementary Table 1. Applications of engineered microenvironments in neuromechanobiology: topography and mechanical stresses (Continued)								
**Topography**	**Random topographies**	Nano-roughness surfaces<br>• R0 (flat): Rq= 1 nm<br>• R1: Rq= 70 nm<br>• R2: Rq= 150 nm	Glass (photolithography and reactive ion etching)	Human ESC	Neural maintenance media	Vinculin; FAK; NMMIIA	• Smooth surfaces (Rq= 1 nm) favoured **self-renewal** (Oct3/4) of human embryonic stem cells.<br>• Rough surfaces (Rq= 70 and 150 nm) promoted **spontaneous differentiation** (loss of Oct3/4).<br>• **Vinculin-containing focal adhesions** were observed on the **periphery of cells** on smooth surfaces (Rq= 1 nm) and throughout the **complete cell area** on nanorough patterns (Rq= 100 nm)	Chen et al., 2012
		Ultra-nanocrystalline diamond (UNCD) film	Diamond (microwave plasma chemical vapour deposition (MPCVD) process)	Rodent neural stem cells	Neural maintenance media	β1-integrin; p-FAK;	• UNCD films **promoted the differentiation of stem cells into neurons** (Tuj-1), **astrocytes** (GFAP) and **oligodendrocytes** (Galc).<br>• **Blocking of β1-integrin prevents cells from differentiating**.<br>• **Increased levels of o-FAK** located at interacting points with UNCD films	Chen et al., 2010
		Nano-roughness surfaces:<br>• R0 (flat, glass): Rq= 3.5 nm<br>• R1-5: Rq= 12 nm<br>• R2: Rq= 16 nm<br>• R3: Rq= 24 nm<br>• R4: Rq= 32 nm<br>• R5: Rq= 80 nm	Silica nanoparticles on glass (spin-coating)<br>Glass (control surface)	PC12 linage; Rodent neural stem cells; Neurons and astrocytes derived from rodent neural stem cells	Neural maintenance media	Piezo-1	• **Neuronal morphology and function** of PC12 cells as well as **neuronal lineage commitment** of rodent neural stem cells were **favoured** at a surface roughness of Rq= 32 nm.<br>• **Interaction between primary rodent neurons and astrocytes** on roughness higher and lower than 32 nm.<br>• These effects were abolished after the **inhibition of Piezo-1**	Blumenthal et al., 2014
	**3D random-porous architectures**	Porous scaffold:<br>• Porosity: 99.5%<br>• Pore size: 100-300 µm<br>• Wall size: 100-200 µm	Graphene (chemical vapor deposition)	Rodent neural progenitors	Neuronal differentiation media	None	• Neural stem cells cultured on porous structures showed an **increased proliferation** (Ki67) **and differentiation towards neuronal** (Tuj-1) and **astroglial** (GFAP) linage compared to cells cultured on graphene films	Li et al., 2013
		Porous scaffold:<br>• Porosity: n.a.<br>• Pore size: n.a.<br>• Wall size: n.a.	Graphene (chemical vapor deposition) and cellulose polymer (bacterial polymerization)	Rodent neural stem cells	Proliferation media / neuronal differentiation media	Vinculin; FAK; Paxilin; β1-integrin	• Neural stem cells showed **higher proliferation** (Ki67, MCM2, PCNA) rates on graphene-cellulose scaffolds.<br>• Graphene-cellulose scaffolds **favoured neuronal differentiation** (Tuj-1) and **activity** (calcium imaging).<br>• **Expression of FA adhesion proteins was lower** in graphene-cellulose than in graphene scaffolds.	Guo et al., 2021

Supplementary Table 1. Applications of engineered microenvironments in neuromechanobiology: topography and mechanical stresses (Continued)								
Topography	3D random-porous architectures	Doughnut-shaped porous scaffold:<br>• Pore size: 500-600µm	Silk-collagen (gelation)	Rodent primary cortical neurons	Neuronal maintenance media	None	• Neuronal bodies located at the porous silk doughnut, while projections grew within a collagen-filled central region	Chwalek et al., 2015
		Porous microspheres:<br>• Porosity: >88%<br>• Pore size: 50 ± 35 µm<br>• Microsphere Ǿ : 100-250 µm	PLGA (emulsion)	iPSC derived NSC	Neural differentiation media	None	• Microspheres promoted differentiation towards both neuronal and glial cell types (Tuj-1, GFAP, NF-M).	Sandhurst et al., 2022
	3D ordered architectures	Circuit of microtowers (Ǿ = 20µm; walls 2,5µ thick) connected through free standing microtubes (Ǿ = 1,5µm; walls 2,5µ thick)	IP-Dip resist (2PP-DLW)	Rodent primary neurons	Neuronal maintenance media	None	• Guided neurite outgrowth along established paths on the scaffold.<br>• Neuronal electrophysiological activity comparable to control substrates.	Fendler et al., 2019
		Circuit of microtowers (Ǿ = 20µm; walls 2,5µ thick) connected through free standing microtubes (Ǿ = 4µm; walls 2,5µ thick)	IP-Dip resist (2PP-DLW)	Human iPSC-derived dopaminergic neurons	Neuronal maintenance media	None	• Guided neurite outgrowth along established paths on the scaffold.<br>• Neurons show electrophysiological activity.	Zierold et al., 2020
		Honeycomb microframe covered on both sides with a monolayer of crossed-linked gelatin microfibers	IP-Dip resist (soft photolithography) and gelatin polymer (gelation)	Human iPSC-derived NSC and brain microvascular endothelial cells (hCMEC/D3 line)	Neural differentiation media	None	• Formation of 3D interconnected neural clusters with high expression of neuronal, astrocytic and synaptic markers.<br>• Spatially correlated neuronal activity.<br>• Astrocyte-endothelium contact.	Huang et al., 2021
		3-layered honeycomb scaffold	DClear resin (2PP-DLW)	Human iPSC-derived NSC	Neural differentiation media	None	• The scaffold enabled long-term culture of iPSC-derived neuron-glia networks composed by neurons of all six cortical layers, different types of interneurons, and astrocytes.<br>• The neural network presented a strong spontaneous neuronal activity.	Koroleva et al., 2021
		Tubular microtowers with or without intraluminal guidance cues (longitudinal micropillars and spiderweb-like platforms) and/or openings.	Ormocomp resin (2PP-DLW)	hESC line derived NSC	Neural differentiation media	None	• The 3D scaffold offers a platform that supports neuronal network formation and neurite orientation.	Turunen et al., 2017

<table>
<tr><td colspan="8">Supplementary Table 1. Applications of engineered microenvironments in neuromechanobiology: topography and mechanical stresses (Continued)</td></tr>
<tr><td rowspan="2">Topography</td><td rowspan="2">3D ordered architectures</td><td>3D nanogrid</td><td>IP-Dip (2PP-DLW)</td><td>Primary rodent neurons</td><td>Neuronal maintenance media</td><td>None</td><td>The scaffold offers a biocompatible platform.
Neurites extend along the fibres with a high degree of alignment to the pattern of the nanogrid</td><td>Agrawal et al., 2021</td></tr>
<tr><td>3D cages decorated with nano and micropillars.</td><td>IP-Dip (2PP-DLW)</td><td>Primary primate microglia</td><td>Microglial culture media</td><td>None</td><td>Microglia present various morphologies when cultured in/on cages.
Microglial colonization of cages is more homogeneous in the presence of nano and micropillars decoration.</td><td>Sharaf et al., 2022</td></tr>
<tr><td rowspan="5">Mechanical stress</td><td>Uniaxial strain</td><td>Elastomeric plates</td><td>PDMS (soft lithography)</td><td>Rat OPC</td><td>Oligodendrocyte differentiation media</td><td>Cytoskeleton</td><td>Strain decreases OPC nuclear fluctuations and cell migration (biophysical markers of differentiation)
Strain-induce increase in tubulin levels, related to OPC differentiation.</td><td>Makhija et al., 2018</td></tr>
<tr><td>Uniaxial strain and Shear stress</td><td>Elastomeric plates (uniaxial strain)
Cultured flask in rotation (shear stress)</td><td>Silicone</td><td>Rat OPC</td><td>Oligodendrocyte differentiation media</td><td>YAP; FA, actomyosin cytoskeleton</td><td>Strain induces the nuclear localization of YAP and assembly of FA.
Shear stress induces a decrease in primary processes mediated by YAP.</td><td>Shimizu et al., 2017</td></tr>
<tr><td>Traction forces</td><td>Pull-up traction assay with integrin-ligand-coated magnetic beads
Tension Gauge Tether Probes</td><td>Glass coverslip</td><td>Rat astrocytic cell line (DITNC1)</td><td>General culture media</td><td>Integrin $\alpha_v\beta3$; Focal Adhesions, actomyosin cytoskeleton</td><td>Mechanical stress increases the number of focal adhesions, stress fibres and surface integrin number in astrocytes.
Cytoskeleton rearrangement generates traction forces via integrins from within the cell.</td><td>Pérez et al., 2021</td></tr>
<tr><td>Uniaxial strain</td><td>Custom-built stretching device</td><td>PDMS</td><td>Human iPSC-derived motor neurons</td><td>Neuronal differentiation media</td><td>None</td><td>Strain induces membrane fluidization of hiPSC-derived motor neurons.
Spontaneous neuronal activity decreases proportionally with increased strain.</td><td>Bianchi et al., 2019</td></tr>
<tr><td>Uniaxial strain</td><td>Custom-built stretching device</td><td>PDMS (soft photolithopgraphy)</td><td>Rat neural stem cells</td><td>Neuronal differentiation media</td><td>None</td><td>Enhanced neurite extension, axon elongation and orientation in the direction of the force
Strain increased neuronal maturation.</td><td>Chang et al., 2013</td></tr>
</table>

Supplementary Table 1. Applications of engineered microenvironments in neuromechanobiology: topography and mechanical stresses (Continued)								
**Mechanical stress**	**Equibiaxial static strain**	Bio-Flex plates: 10% static equibiaxial stretch	Rubber	Embryonic rodent NSC	Differentiation media	Integrins	• Static stretch **decreases oligodendrocyte** differentiation on laminin-coated membranes, an effect mediated by **integrin** binding.<br>• Static stretch did not affect neuronal and astrocytic differentiation.	Arulmoli et al., 2015
	**Traction force microscopy**	L1-CAM-Fc (adhesion protein) coated acrylamide gels with embedded 200-nm fluorescent beads	Acrylamide (gelation)	Primary rodent neurons	Neuronal maintenance media	Shootin1b	• Traction forces on the growth cone induce its advance.<br>• This is mediated by Shootin1b	Minegishi et al., 2018
	**Biaxial cyclic strech**	Fexcell plates Flexcell FX-5000 T tension system	Rubber	Primary rodent astrocytes	Astrocyte maintenance media	Cytoskeleton	• Mechanical stretch caused cytoskeleton rearrangement of astrocytes.<br>• Under mechanical stretch, changes in protein expression and signalling pathways associated with astrocytic activation can be observed.	Li et al., 2022
	**Uniaxial cyclic stretch**	Stretching device (CellScale MechanoCulture T6)	PDMS	Neuron lineage differentiated PC12 cells (rat)	Neural maintenance media	Cytoskeleton	• PC12 orient perpendicular to the direction of the stretch.<br>• Stretch does not influence the outgrowth of neurite of PC12 cells.	Lin et al., 2020
	**Uniaxial stretch**	Custom-built stretching device	PDMS (soft lithography)	Human iPSC-derived neurons	Neuronal maintenance media	None	• Stretch stimulates rapid amyloidogenic processing of the amyloid precursor protein	Chaves et al., 2021

Supplementary Table 1. Applications of engineered microenvironments in neuromechanobiology: topography and mechanical stresses. *Abbreviations.* PDMS: Polydimethylsiloxane; PLGA: poly(lactic-co-glycolic acid); 2PP-DLW: direct laser writing by two-photon polymerization; iPSC: induced pluripotent stem cell; ESC: embryonic stem cell; MSC: mesenchymal stem cell; NSC: Neural stem cell; OPC: oligodendrocyte precursor cell.

Supplementary Table 2. Applications of engineered microenvironments in neuromechanobiology: stiffness and viscosity

Mechanical property or stimulation	Biological outcome	Substrate features	Material and fabrication technique	Cell type	Culture media	Mechanistic study	Biological outcome description	Ref.
Stiffness	Cell differentiation and maturation	100, 700, and 75 000 Pa	Polyacrylamide gel (gelation)	Human iPSC and ESC	Neuronal differentiation media	None	• Increased **neuroectodermal and neuronal differentiation** (PAX6, TUJ1) in softer substrates (**100 and 700Pa**)	Keung et al., 2012
		100-10 000 Pa	Hydrogel (gelation)	Rat adult NSC	Neuronal differentiation media / Astrocyte differentiation media	None	• Increased **neuronal differentiation** (TUJ1) in soft substrates (**500Pa**)<br>• Decreased **astrocyte survival** in soft substrates (GFAP)	Saha et al., 2008
		100 - 75 000 Pa	Polyacrilamide gels (gelation)	Rat adult NSC	Mixed (neuronal and glial) differentiation media	RhoA and CDC42 activation; actomyosin contractility	• Increased **neuronal differentiation** (TUJ1) in relatively soft substrates (**100-700Pa**)<br>• **Equal proportion of neurons** (TUJ1) **and astrocytes** (GFAP) in stiffer substrates **(1500-75000Pa).**<br>• **Increased cellular stiffness** in stiffer substrates	Keung et al., 2011
		0.7 and 750 kPa	Qgel (silicone elastomer)-based substrates (gelation)	Primary human NPC	Neuronal differentiation media	Piezo 1 and YAP activation	• **Piezo 1**-mediated Ca2+ transients **increase** on stiffer substrates (750 kPa).<br>• Increased **neuronal differentiation** (MAP2) in stiffer substrates (750 kPa).<br>• Increased **astrocytic differentiation** (GFAP) after Piezo1 pharmacological or genetic inhibition.<br>• **Increase cytoplasmic location of YAP** on soft (0.7kPa) substrates.	Pathak et al., 2014.

Supplementary Table 2. Applications of engineered microenvironments in neuromechanobiology: stiffness and viscosity (Continued)								
**Stiffness**	**Cell differentiation and maturation**	*Topography:*<br>• *Flat structures:* 0.5kPa, 5kPa and 2.5MPa<br>• *Micropost arrays (1.8 and 0.8 µm):* 1, 3 and 1200kPa	PDMS (photolithography and replica moulding)	Human ESC	Neuronal differentiation media	YAP/TAZ; Hippo pathway; actomyosin cytoskeleton, RhoA	• Increased **neuroectodermal and neuronal differentiation** (PAX6, TUJ1) in soft (**≤5 kPa**) microarrays, regardless of their diameter.<br>• Increased **neuroectodermal** (PAX6) in soft (**0,5 and 5 kPa**) flat structures, but less drastic than microarrays.<br>• **Posterior and anterior patterning genes** (OTX2, HOXB1, HOXB4, and HOXC8) **are responsive to varying** substrate **stiffness** (**5 vs 1200kPa**) in the presence of anterior/posterior patterning cues (Pur, RA).<br>• **Increase YAP and Smad** localization on the cytoplasm on soft substrates.<br>• **Hippo**-pathway mediated **phosphorylation** of **YAP** on soft substrates<br>• **Actin microfilaments bundled or diffusely distributed** in hESC cultured on stiff or soft substrates, respectively.	Sun et al., 2014
		0.1–1 kPa and ∼ 50–100 kPa	Polyacrylamide gel (gelation)	Rat BMMSC	Maintenance media	β1 integrin	• Enhanced β1 integrin **activation and internalization** on soft substrates<br>• Increase **neurogenic differentiation** on soft substrates<br>• **Reduced phosphorylation of Smads** 1/5/8 on soft substrates.	Du et al., 2011
		~0.7kPa and ~10kPa	Polyacrylamide gel (functionalized with peptides) (gelation)	Human ESC	Pluripotency maintenance media	YAP/TAZ; actomyosin cytoskeleton	• Increased **neuronal differentiation** (TUJ1) in soft substrates (**0.7kPa**) as a consequence of **cytoplasmic** localization of **YAP** and **decreased F-actin polymerization**	Musah et al., 2014
		2-3000 MPa<br>*Topography: fibers of 1.5 and 1.0 µm*<br>*Technique: electrospinning*	PCL, PLA, gelatin (electrospinning)	Rat primary OPC	Myelination media	YAP	• **Increased** oligodendrocyte **differentiation** but **decreased myelination** in stiff substrates<br>• YAP regulates the mechanotransduction pathway leading to **myelination** *but not differentiation.*	Ong et al., 2020

Supplementary Table 2. Applications of engineered microenvironments in neuromechanobiology: stiffness and viscosity (Continued)								
**Stiffness**	**Cell differentiation and maturation**	2.5, 6.5 and 10 kPa	Polyacrylamide gel (gelation)	Rat primary OPC	Oligodendrocyte differentiation media.	None	• **Increased** oligodendrocyte **differentiation and maturation** (CTCF, MBP, PLP) in soft (6kPa) substrates	Lourenço et al., 2016
		0.1 to 70 kPa	Polyacrylamide gel (gelation)	Rodent OPC	Proliferation media / Oligodendrocyte differentiation media	None	• OPCs **stiffen** during **differentiation** independently of substrate stiffness.<br>• Enhanced oligodendrocyte **differentiation** (MBP) with increased stiffness substrates.	Jagielska et al., 2012
		1.5 and 30 kPa	Polyacrylamide gel (gelation)	Rat primary OPC	Proliferation media / Oligodendrocyte differentiation media	NMII<br>Myosin light chain<br>YAP,<br>Lamin A/B	• **Decrease** oligodendrocyte **differentiation and maturation** (Olig1) in stiff (30kPa) substrates.<br>• **Increase** in levels of **phosphorylated myosin light chain and NMII activity** in stiff (30kPa) substrates.<br>• **Increased nuclear** localization of **YAP** on stiff (30kPa) matrices.<br>• **Reduced** expression of **type B lamin** and **increased** expression of **lamin A** on stiff (30kPa) matrices	Urbanski et al., 2016
	**Cell function and morphology**	*Topography:*<br>• *Flat structures:* 0.5kPa, 5kPa and 2.5MPa<br>• *Micropost arrays (1.8 and 0.8 µm):* 1, 3 and 1200kPa	PDMS (soft lithography)	Human ESC	Neuronal differentiation media	None	• Motor neurons derived from soft PMAs exhibit **electrophysiological activities comparable to those from primary neurons *in vivo***	Sun et al., 2014
		~0.7kPa and ~10kPa	Polyacrylamide gel (functionalized with peptides) (gelation)	Human ESC	Pluripotency maintenance media	None	• Neurons differentiated in soft substrate show spontaneous **postsynaptic currents** and **action potentials** earlier than those cultured by standard protocols (only biochemical cues).	Musah et al., 2014
		Stiffness gradient from 520 Pa to 3.41 kPa	Polyethylene glycol hydrogel (gelation)	Human iPSC-derived NSC	Neural differentiation media	None	• hNSCs extend **longer neurites** in soft (907Pa) substrates.<br>• No differences in TUJ1 or MAP2 expression were found across stiffness.	Mosley et al., 2017

Supplementary Table 2. Applications of engineered microenvironments in neuromechanobiology: stiffness and viscosity (Continued)								
**Stiffness**	**Cell function and morphology**	2 kPa to 125 kPa (polyacrylamide 2D gels)<br>260 and 829 Pa (collagen I 3D gels)	2D polyacrylamide gels and 3D collagen I gels (gelation)	Human iPSCs-derived forebrain and motor neurons	Motor neuron differentiation media or forebrain neuron differentiation media	RHOA; Myosin II; FAK, Src	PAA 2D gels<br>• hIPSC-derived motor neurons extend **longer neurites** and do it **faster** on stiff substrates.<br>• hIPSC-derived forebrain neurons, neurite extension is **faster** on soft substrates, but their **length does not differ** across rigidities.<br>• **Increased activity of RHOA and myosin II, FAK and Src** on motor neurons on stiff substrates.<br>Collagen I 3D gels<br>• hIPSC-derived motor neurons extend **longer neurites** on stiff substrates, while hIPSC-derived forebrain neurons do it on softer ones.	Nichol IV et al., 2019
		42.7; 307.6 and 990.6 kPa	Alginate and collagen type I hybrid gels (gelation)	Primary rat astrocytes	Glial media	YAP; F-actin	• On soft substrates, astrocytes present a **naïve** state, with **round** morphology and **low expression** of astrocyte markers (GFAP and IL-1β).<br>• On stiff substrates, astrocytes present a **reactive phenotype**, with a **hypertrophic and extended** morphology and **upregulated expression** of astrocyte markers.<br>• Astrocytic phenotype can be switched by modulating matrix stiffness.<br>• **Higher expression** and **nuclear localization** of **YAP** on astrocytes cultured on stiff substrates.	Hu et al., 2021
		1 kPa, 4 kPa, and 11 kPa	Polyacrylamide gel (gelation)	Human astrocyte cell line	Astrocyte media	Cell-substrate tractions;<br>Strain energies;<br>Cell-cell intercellular stresses;<br>Cellular velocities	• Astrocytes **elongate** with increased substrate stiffness.<br>• **Increased tractions, strain energy and intracellular stress but decreased cellular velocities** are observed with increasing substrate stiffness	Bizanti et al., 2021
		0.1 to 70 kPa	Polyacrylamide gel (gelation)	Human iPSC-derived oligodendrocytes	Oligodendrocyte differentiation media	None	• **Increased migration** of hiPSC-derived oligodendrocytes with increased stiffness	Espinosa-Hoyos et al., 2020

Supplementary Table 2. Applications of engineered microenvironments in neuromechanobiology: stiffness and viscosity (Continued)								
**Stiffness**	**Cell function and morphology**	0.6 kPa, 1 kPa and 1.2 MPa	PDMS (gelation)	Rat primary microglia	Glial media	Stretch-Dependent Cl−Channels	• **Increased** microglia **proliferation** (BrdU) in soft substrates.<br>• Microglia exhibit a **round** morphology (Iba1) with increased substrate stiffness.<br>• **Enhanced** microglia **anti-inflammatory activation** on soft substrates.	Blaschke et al., 2020
		100 Pa, 10 kPa and 30 kPa	Polyacrylamide gel (gelation)	Rat primary microglia and astrocytes	Glial media	None	• Microglia cells exhibit a **spherical** morphology and short processes on soft (100Pa) substrates, while they spread and present **extended processes** in stiff (30kPa) substrates (characteristic of an activated phenotype).<br>• Astrocytes show a **spherical** or **star-like** morphology with **fine processes** on soft (100Pa) substrates, while they present a spread **polygonal shape** on stiff (30kPa) substrates (characteristic of an activated phenotype).<br>• Astrocytes and microglia cells upregulate inflammatory genes and proteins in in stiff (30kPa) substrates.	Moshayedi et al., 2014
		Cell force measurement: stiffness gradient of 10 Pa and 10kPa.<br><br>Migration studies: 100 Pa, 300 Pa, 1 kPa, 10 kPa	Polyacrilamide gel (gelation)	Rat primary microglia	Glial media	None	• Microglia cells exert **forces** that **increase** with substrate stiffness and reach a plateau at 5 kPa of substrate stiffness.<br>• Microglia **migrates preferentially** towards stiff substrates.	Bollmann et al., 2015
**Viscosity**	**Cell differentiation and morphology**	Half relaxation time: 1200 to 60 s	Hyaluronan hydrogels (gelation)	Neuron lineage differentiated PC12 cells (rat)	Neuronal differentiation media	Vinculin; Piezo 1; YAP	• **Enhanced neurogenesis and axonal elongation** with faster matrix relaxation times.<br>• Faster stress relaxation leads to **larger FAs**, **decreased Piezo 1 expression** and **cytoplasmic location of YAP**.	Chen et al., 2021
		Relaxation rate of 0.1-0.2 s-1	Collagen/hyaluronic acid hydrogel (gelation)	Primary human fetal-derived astrocytes	Astrocyte differentiation media	None	• **Branched morphology** and **lowest GFAP** level expression on gels with a relaxation rate of **0.1-0.2 s-1**, suggesting a quiescent state.	Placone et al., 2015

Supplementary Table 2. Applications of engineered microenvironments in neuromechanobiology: stiffness and viscosity. *Abbreviations*. PDMS: Polydimethylsiloxane; PCL: Polycaprolactone; PLA: poly(lactic acid); iPSC: induced pluripotent stem cell; ESC: embryonic stem cell; BMMSC: Bone marrow mesenchymal stem cell; NSC: Neural stem cell; OPC: oligodendrocyte precursor cell.
